# *ARX* mutation-associated interneuron defects provide insights into mechanisms underlying developmental epilepsies

**DOI:** 10.1093/brain/awag036

**Published:** 2026-02-03

**Authors:** Youngshin Lim, Shyam K Akula, Abigail K Myers, Connie Chen, Katherine A Rafael, Michaela G Ibach, Edwin Trevathan, Christopher A Walsh, Jeffrey Golden, Ginam Cho

**Affiliations:** Department of Pathology and Laboratory Medicine, Cedars-Sinai Medical Center, Los Angeles, CA 90048, USA; Department of Biomedical Science Education, Charles R Drew University of Medicine and Science, Los Angeles, CA 90059, USA; Division of Genetics and Genomics, Manton Center for Orphan Disease Research, Boston Children’s Hospital, Boston, MA 02115, USA; Departments of Pediatrics and Neurology, Harvard Medical School, Boston, MA 02115, USA; Howard Hughes Medical Institute, Chevy Chase, MD 20815, USA; Department of Psychology, Hamilton College, Clinton, NY 13323, USA; Department of Pathology and Laboratory Medicine, Cedars-Sinai Medical Center, Los Angeles, CA 90048, USA; Department of Pathology and Laboratory Medicine, Cedars-Sinai Medical Center, Los Angeles, CA 90048, USA; Section of Palliative Care, Division of Hospital Medicine, Department of Pediatrics, Vanderbilt University Medical Center, Nashville, TN 37232, USA; Departments of Pediatrics and Neurology, Vanderbilt University Medical Center, Vanderbilt Institute for Global Health, Nashville, TN 37232, USA; Division of Genetics and Genomics, Manton Center for Orphan Disease Research, Boston Children’s Hospital, Boston, MA 02115, USA; Departments of Pediatrics and Neurology, Harvard Medical School, Boston, MA 02115, USA; Howard Hughes Medical Institute, Chevy Chase, MD 20815, USA; Department of Pathology and Laboratory Medicine, Cedars-Sinai Medical Center, Los Angeles, CA 90048, USA; Department of Pathology and Laboratory Medicine, Cedars-Sinai Medical Center, Los Angeles, CA 90048, USA

**Keywords:** transcription factor, inhibitory neuron, gene regulatory network, neural differentiation, cortical entry

## Abstract

Cortical interneuron (cIN) dysfunction is associated with various neurodevelopmental and neurological disorders, including developmental epilepsies, autism spectrum disorders and intellectual disabilities. Mutations in *ARX* (aristaless-related homeobox) are linked to these conditions, with or without accompanying structural brain anomalies. We previously demonstrated that the loss of *Arx* in the mouse ganglionic eminence, the birthplace of cINs, is associated with seizures, whereas its loss in cortical excitatory neuron progenitor cells results in structural anomalies but no seizures.

To elucidate the pathophysiological role of ARX in cINs and its relationship to seizure phenotype, *Arx* conditional mutant mouse lines were investigated using *Gad2*- and *Nkx2.1*-Cre drivers to target distinct populations in the cIN lineage.

Our data demonstrate that ARX abrogation results in defects in cIN density and distribution, as well as perinatal lethality. In these mice, we observed defects in cell cycle exit, a biased loss of the marginal zone migration stream of cINs, shifts in cell fate from caudal ganglionic eminence to medial ganglionic eminence identity, and a reduced number of parvalbumin⁺ and somatostatin⁺ cINs, with parvalbumin⁺ cINs being more severely affected. Single-cell RNA sequencing combined with chromatin immunoprecipitation and sequencing revealed that ARX regulates key processes involved in cell cycle progression, cIN subtype differentiation and cIN migration.

Investigation of one downregulated target gene, *Lmo1*, uncovered a potential mechanism by which ARX regulates the number and distribution of cINs in the cortex. Cortical slice cultures demonstrate that LMO1 inhibits cIN migration by repressing *Cxcr4* expression, which encodes a key receptor involved in cortical guidance. These data indicate that ARX positively regulates cIN migration by derepressing LMO1's repressive role. Consistent with our mouse model, we observed a significant loss of parvalbumin+ and somatostatin+ cINs in the brain of a patient carrying a pathogenic variant of *ARX*, who was diagnosed with developmental epileptic encephalopathy.

Together, our data provide novel insights into how ARX and its target genes regulate cIN development and migration and into the pathogenic mechanisms underlying a spectrum of neurodevelopmental disorders linked to loss of ARX.

## Teaser

Lim *et al.* show that loss of the transcription factor ARX disrupts cortical interneuron development and migration. Using conditional mutant mouse lines, they identify ARX-regulated pathways controlling interneuron fate and distribution, and demonstrate comparable interneuron deficits in a patient with a pathogenic *ARX* variant.

## Introduction

Developmental epileptic encephalopathies (DEEs) are a group of severe epilepsies characterized by early-onset seizures and developmental disorders, including intellectual disabilities, autism spectrum disorders and/or loss of motor skills.^1,2^ DEEs typically present in infancy or early childhood and are marked by frequent, treatment-resistant seizures.^1,2^ Examples of DEEs include early infantile epileptic encephalopathy (including Ohtahara syndrome), early myoclonic encephalopathy and infantile epileptic spasms syndrome (including West syndrome). These conditions are frequently linked to mutations in a variety of genes, including the aristaless-related homeobox (*ARX*) gene (NM_139058; MIM# 300382).^3^ Mutations in *ARX* are associated with a broad spectrum of phenotypes, ranging from mild intellectual disability without structural brain abnormalities to severe DEEs accompanied by brain structural anomalies.^4^

Accumulating evidence suggests that an interneuron defect may underlie the epilepsy phenotype observed in DEE patients, including those with *ARX* mutations.^5^ In fact, perturbations in cortical interneuron (cIN) development and function have been implicated not only in developmental epilepsies but also in intellectual disabilities, autism spectrum disorder and schizophrenia.^6-8^ Thus, understanding the mechanisms that control the differentiation, migration and circuit integration of cINs is critical for the development of biologically based targeted therapies for these disorders referred to as interneuronopathies.^5-8^

Approximately 60%–70% of cINs originate from the medial ganglionic eminence (MGE) and express parvalbumin (PV) or somatostatin (SST),^9-11^ while around 30% arise from the caudal ganglionic eminence (CGE) and 10% or fewer from the preoptic area (POA).^10-13^ In humans, a minor subset of cINs is derived from pallial progenitors.^14,15^ Recent advances in transcriptomics have identified over 50 subtypes of cINs.^16^ Transcriptomic studies, along with classical transcription factor expression mapping studies, suggest that cIN diversity emerges early in the proliferative regions of the MGE and CGE, ventricular zone (VZ) and subventricular zone (SVZ), before the progenitor cells become post-mitotic and differentiate.^17-19^

Another important feature of cINs is their complex migratory pathways.^9,11,20,21^ From their birthplace, they migrate along defined paths to the developing cortex. Migration is initiated by chemorepulsive signals from the proliferative zones of the ganglionic eminences (GEs).^20,21^ Cortically directed interneurons rely on NRP1/SEMA3A-mediated repulsion to navigate around the developing striatum and respond to chemoattractant cues such as CXCL12 and NRG1 to guide their migration toward the cortex.^20-22^ Upon entering the dorsal telencephalon, cINs are parsed into three migratory streams: the marginal zone (MZ), intermediate/subventricular zones (IZ/SVZ) and subplate (SP).^20^ How specific routes are selected remains incompletely understood.

While previous studies have reported reductions in select populations of cINs following *Arx* deletion in mice,^23^ the underlying mechanisms remain poorly understood. To address this gap, we investigated a series of *Arx* mutant mouse models and the associated transcriptional networks involved in cIN development. These models include conditional *Arx* knockouts using IN-specific *Cre* lines, as well as a mouse line carrying human disease-associated polyalanine tract expansion in *Arx.*^24^ Our data reveal that ARX and its downstream target genes regulate key processes during cIN development, including the transition from proliferation to differentiation, subtype specification and migration along the MZ stream. Analysis of brain tissue from a patient with DEE carrying a pathogenic *ARX* variant supports our findings on subtype specification. Moreover, we show that ARX normally promotes IN migration to the cortex by directly repressing *Lmo1*, which itself acts as a transcriptional repressor of a critical guidance receptor, *Cxcr4*. Together, these findings offer new insights into the mechanisms governing cIN development and migration, provide additional evidence for the role played by cINs in DEEs, and further explain the broad spectrum of overlapping phenotypes observed in patients with *ARX* mutations.

## Materials and methods

### Mice

All procedures and animal care were reviewed, approved and performed in accordance with the Cedars-Sinai Medical Center Institutional Animal Care and Use Committee guidelines. Mouse strains (Jackson Laboratory) used in this study include *Rosa^Ai14/Ai14^* (Rosa 26-LSL-tdTomato Cre-reporter) (Stock No. 007914),^25^  *Gad2Cre* (Stock No. 028867),^26^  *Nkx2.1-CreER* (Stock No. 014552),^26^  *Nkx2.1-Cre* (Stock No. 008661),^27^  *SstCre* (Stock No. 013044),^26^  *Dlx5/6-Cre-Gfp*,^28^  *Arx-flox*,^29^  *Arx*^(GCG)7^ (Arx^PA1–22^)^24^ and *Cxcr4-flox* (Stock No. 008767).^30^ For timed pregnancies, the day of the vaginal plug was designated as embryonic Day (E)0.5. For tamoxifen induction, 3.75 mg of tamoxifen was administered to pregnant dams by intraperitoneal (i.p.) injection (150 ml of 25 mg/ml stock plus 150 ml of corn oil) and caesarean section was performed at E19.5–20.5 for postnatal Day (P)0 harvest.

### Single-cell RNA sequencing, differentially expressed genes and gene set enrichment analyses

E16.5 forebrains were harvested and incubated with papain solution (Worthington Biochemical) for 20 min at 37°C. After gentle trituration, tdTomato+ cells were isolated by cell sorter (BD FACSymphony S6) and loaded onto a 10x Genomics single-cell preparation platform. A minimum of 5000 cells were recovered per sample [two wild-type (WT) and three mutant males]. Library preparation was performed using 10x Chromium Single 3′ reagent kits, version 2 (10x Genomics). The library length and concentration were quantified using a Bioanalyzer (Agilent). Paired-end sequencing was performed on the Illumina NovaSeq. FASTQ files were aligned with mouse genome (mm10) by Cellranger V6.0 (10x Genomics). The scvi-tools package^31^ was used for normalization, removal of doublet, dimensionality reduction, integration across biological conditions, differential expression (DE) and Uniform Manifold Approximation and Projection (UMAP) visualizations. Gene set enrichment analysis (GSEA) was performed using the FunRich package,^32^ filtering DE genes at *P*_adj_ < 0.05 and |fold-change| > 1.5. R package CellChat^33^ was used for cell-cell communication analysis with default parameters; Python package CellOracle^34^ for comparing gene regulatory network models; and Gephi^35^ to visualize networks.

### Microarray analysis and next-generation sequencing transcriptome data analysis

cDNAs were amplified from extracted RNAs using the QIAGEN RNAeasy kit after purification of GFP-positive interneurons from the forebrain of E14.5 *Arx*^(GCG)7/Y^ and WT littermate controls crossed with *Dlx5/6-Cre-IRES-Gfp* mice. For each microarray, 1 μg of cDNA was labelled and hybridized to an Affymetrix Mouse Whole Genome 430 2.0 Array, with six independent replicates per condition. The Bioconductor software package was used to background-correct and normalize the data and calculate mean fold-change (FC) and false discovery rates (FDRs) for all pair-wise comparisons.

### Chromatin immunoprecipitation and sequencing

E14.5 forebrains were isolated and fixed in 4% paraformaldehyde (PFA) in phosphate buffered saline (PBS) for 16 h at 4°C. Fixation was quenched with 125 mM glycine at room temperature (RT) for 5 min and washed with cold PBS twice. Chromatin immunoprecipitation (ChIP) was performed using Simplechip^®^ Plus Sonication Chromatin IP Kit (Cell Signaling Technology) with minor modifications. First, the forebrain tissue was soaked with citrate antigen retrieval buffer and heated for 15 min by double boiling, then washed with nuclease-free H_2_O and neutralized with the ChIP buffer. Chromatin was fragmented into 100–300 bp fragments using the Bioruptor UCD-200 (Diagenode; high power) for 2 h (cycled at 30 s ON/30 s OFF). For IP of ARX-bound chromatin, 2 μg of anti-ARX antibody (R&D) was incubated with cleared chromatin lysate. Input and ChIPed DNA libraries were prepared using an Illumina Next Seq (single-end reads of 75 bp). FASTQ sequences were aligned to the mouse mm10 genome sequence using HISAT2 and converted to SAM and then BAM files. ChIP sequencing (ChIP-seq) peaks were called using MACS2 (input DNA without ChIP as reference using default settings). The HISAT2-aligned peaks and MACS-determined peak positions were visualized using the IGV Genome Browser.

### Tissue immunolabelling

Embryonic brains were harvested and fixed in 4% PFA overnight at 4°C. Postnatal brains were harvested after trans-cardiac perfusion (PBS with 60 mM of heparin and then 4% PFA). Harvested brains were used for cryosection (14 µm) and subsequently for immunofluorescence labelling as previously described.^36^ Slides were incubated with primary antibodies overnight at 4°C, then incubated with biotinylated or fluorescence-conjugated secondary antibodies for 1 h at RT. Primary antibodies included rabbit anti-RFP (1: 500, Rockland), rabbit anti-CXCR4 (1: 200, Abcam), rat anti-somatostatin (1: 50, Scbt), mouse anti-PV (1: 250, Swant), rabbit anti-ERBB2/4 (1:1000, Abcam), and rabbit anti-DLX2 (1:200, gift from Dr David D. Eisenstat at the University of Melbourne). Secondary antibodies conjugated to Alexa-488, -594, or -647 fluorophores were all used at 1:250 (ThermoFisher). Sections were mounted with Prolong Glass Mounting Media (Invitrogen, P36984) containing DAPI (Vector Labs). For DAB-based immunolabelling, the ABC kit (Vector Lab) and detection system were used according to the manufacturer's recommendation.

### Cell cycle analyses

5-Ethynyl-2′-deoxyuridine (EdU; Invitrogen) was administered i.p. (50 mg/kg) to pregnant mice (E13.5 for 1.5, 24 or 48 h). Click-iT EdU cell proliferation kit (Invitrogen) was used to label EdU+ cells. Cells in the active cell cycle were labelled with a Ki67 (D3B5) antibody (1:200 dilution, Cell Signaling Technology) as described earlier.

### RNAscope

Cryosections (E14.5), 16 µm thick, were prepared as previously described.^36^ Sections were air-dried at 60°C for 1 h, followed by 4% PFA post-fixation at 4°C for 15 min. Target retrieval was performed by double boiling the sections for 7 min and treating with Protease III for 10 min. *In situ* hybridization was performed following the manufacturer's (Advanced Cell Diagnostics, Inc.) protocol for the multiplex fluorescent kit (v.2) (for mouse brains) or RNAscope^®^ 2.5 HD Detection Reagent—BROWN (for human brains). RNAscope probes were Mm-Maf (Cat. No. 412958), Mm-*Mef2c* (Cat. No. 421011) and Hs-SST (Cat. No. 310591). Slides were mounted with Prolong glass mounting media (Invitrogen, Cat. No. P36984) containing DAPI.

### Tissue clearing, immunolabelling, and cell counting

Tissue clearing was performed using the SHIELD protocol by LifeCanvas Technologies.^37,38^ Samples were cleared for 7 days with Clear+ delipidation buffer, immunolabelled using SmartLabel [5 μg mouse anti-NeuN (RRID:AB_2572267), 4 μg rabbit anti-PV (Swant PV27; 1:400) and rat anti-somatostatin (Millipore), followed by fluorescent secondary antibodies], and then incubated in EasyIndex for a refractive index of 1.52 and imaged at ×3.6 using SmartSPIM microscopy. Images were tile-corrected, de-striped and registered to the Allen Brain Atlas. The NeuN channel was registered to the 8–20 atlas-aligned reference samples using successive rigid, affine and B-spline warping (SimpleElastix). An average alignment to the atlas was computed across all intermediate reference-sample alignments to serve as the final atlas alignment value per sample. Fluorescence measurements from the acquired images were projected onto the Allen Brain Atlas to quantify the total fluorescence intensity per region defined by the Allen Brain Atlas. These values were then divided by the corresponding regional volume to calculate the intensity per voxel. Data were plotted using Prism (v.10).

### Slice culture and electroporation

CD-1 embryos were collected on E14.5. The brains were harvested and embedded in 3% low melting point agarose (Fisher Scientific, Cat. No. BP165-25) and sectioned at 300 μm (Leica VT 1000s).^39,40^ pCIG or pCIG-Lmo1 plasmids were micropipetted into the medial ganglionic eminence of each slice followed by electroporation (Nepa Gene CUY21 electroporator; 60 V, pulse interval: 5 ms, pulse duration: 200 ms, number of pulses: 5). Slices were cultured on laminin and poly-L-lysine coated transwell inserts (BD Falcon, Cat. No. 353102) for 3 days at 37°C and 5% CO_2_,^41^ after which slices were fixed with 4% PFA overnight at 4°C.

### Explant culture and cell migration analysis

Embryonic Day 14.5 MGEs were dissected in ice-cold Hank’s balanced salt solution (Sigma) and used for explant culture as described previously.^39^ The explants were imaged on a Zeiss Observer Z1 inverted microscope.

### Image analyses and quantifications

For cell density determination, coronal sections were taken from the septum to the CGE. The entire cortical area spanning the dorso-medial apex to the pallial-subpallial boundary was taken as the region of interest (ROI). The total numbers of tdTomato+, PV+, SST+ and DLX+ cells in this ROI were counted manually in ImageJ using the StarDist 2D plug-in and divided by the area of the ROI (in mm^2^). Cortical IN distribution was established using the entire pallium divided into 10 bins from the ventricular surface (bin 1) to the pial surfaces (bin 10), and a single counting field (200 µm wide for E14.5 and 400 µm wide for P0) per section was used. For quantification of the EdU injection experiments, the GE-cortical boundary area was taken as the ROI on multiple levels of coronal sections covering the septum to the CGE. Using the StarDist 2D plug-in, each DAPI+ cell was set per ROI, and the numbers of EdU+ or Ki 67+ cells were counted.

### Human patient information

A term male, born to a mother with treated hypothyroidism and an otherwise uncomplicated pregnancy and delivery, presented with abnormal movements at 5 months of age. His development was normal through 4 months, with a past medical history significant only for gastroesophageal reflux and strabismus. Ophthalmologic exam at 4 months noted delayed visual maturation with minimal reaction to light and congenital exotropia. A brain MRI was normal at 5 months of age. EEGs were recorded during waking and sleep multiple times, using the standard international 10–20 scalp electrode placement system. The initial EEG, at 5 months of age, showed hypsarrhythmia, and he was diagnosed with infantile spasms. Genetic evaluation was based on IDT X-gene exome sequencing (Integrated DNA Technologies). The library products were sequenced with 2 × 150 bp reads on an Illumina NovaSeq. Reads were aligned to the human genome build GRCh37/UCSC hg19 and analysed for sequence variants using several variant calling algorithms, including ones developed in the Genosity Lab, and a pathogenic variant of *ARX* (p. Arg384His; c.1151G>A in exon 4) was identified (this variant has been reported in a Chinese cohort with infantile spasms).^42^ He subsequently developed intractable symptomatic multifocal epilepsy with a declining quality of life and died at age 22 months. Consent for post-mortem examination and utilization of biological samples, along with clinical information, for research was obtained from the parents and approval for human subject research through the Cedars Sinai Medical Center Review Board. Immunofluorescence labelling and RNAscope analyses were performed using the same methods as described for the mouse tissues. Control brain sections were from a 24-month-old male with a history of metastatic neuroblastoma and no history of seizures.

### Statistical analyses

GraphPad Prism was used for all statistical analyses. A two-tailed *t*-test (with Welch’s correction) was used for data with normal distribution; a non-parametric test (Mann-Whitney test or Kolmogorov-Smirnov test) for data with non-normal distribution. All samples were randomly selected, and image quantifications were carried out blindly. *P*-values ≤0.05 were considered statistically significant. All bar graphs are plotted as mean ± standard error of the mean or mean ± standard deviation, and all scattered plots are marked with individual data points and median with interquartile range.

## Results

### Loss of *Arx* results in abnormal numbers and distribution of cortical interneurons

**Figure 1:**
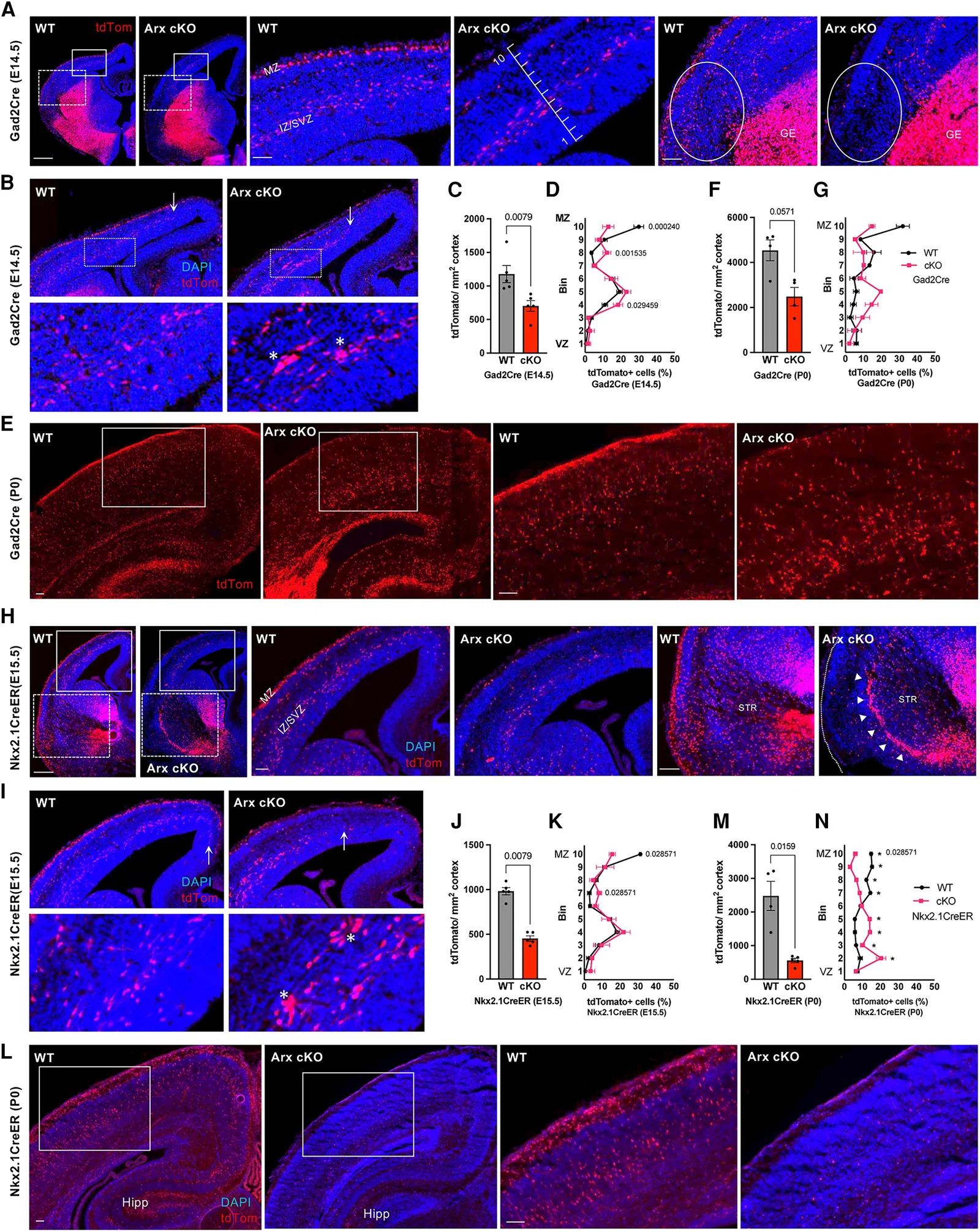
**Cortical interneuron density and distribution defects in *Arx* conditional knockout mice** (**with *Ai14***) **driven by *Gad2Cre* and *Nkx2.1CreER*.** Representative images of tdTomato (red) on coronal sections from wild-type (WT) and *Arx* conditional knockout (cKO) brains under *Gad2Cre* (**A**, **B** and **E**) and *Nkx2.1CreER* (**H**, **I** and **L**), taken at embryonic Day (E)14.5–15.5 (**A**, **B**, **H** and **I**) or postnatal Day (P)0 (**E** and **L**). DAPI (blue) was used for nuclear staining. (**A** and **H**) *Middle two panels*: Magnified images of the lined boxed areas in the *first two panels*. *Right two panels*: Magnified images of the dotted boxed areas in the *first two panels*. Ten equally distributed bins are marked with white lines. Circled areas are examples of the cortical entry area of the interneurons (INs). White arrowheads in **H** indicate accumulated clusters of tdTomato+ INs at the striatal boundary. (**B** and **I**) *Top*: Different sections from the same brains in **A** and **H**, respectively. *Bottom*: Magnified images of the boxed areas in the *top* panels. Arrows indicate the front of the intermediate/subventricular zone (IZ/SVZ) migration stream; asterisks = clustered cells; dotted line = the boundary of the section. (**E** and **L**) *Right two panels*: Magnified images of the *left two panels*. (**C**, **D**, **F**, **G**, **J**, **K**, **M** and **N**) Quantification of tdTomato+ cell density (**C**, **F**, **J** and **M**) and the percentage of the tdTomato+ cells in each bin to the total tdTomato+ cell numbers in all 10 bins (**D**, **G**, **K** and **N**) in WT and cKO neocortices at E14.5–15.5 (**C**, **D**, **J** and **K**) or P0 (**F**, **G**, **M** and **N**). *n* = 4 or 5 mice per genotype. Each data point in **C**, **F**, **J** and **M** represents the average of 3–4 tissue sections of each mouse. Mann-Whitney test was used in **C**, **F**, **J** and **M**; Multiple Mann-Whitney tests in **D**, **G**, **K** and **N** (*n* = 16 sections from four mice per genotype). Error bars in each graph: mean ± standard error of the mean. Numbers over the bars or next to data points: *P*-values. All asterisks in **N** denote the same *P*-values as indicated. Scale bars in **A** = 400 µm; all others = 100 µm. CP = cortical plate; GE = ganglionic eminence; Hipp = hippocampus; IZ = intermediate zone; MZ = marginal zone; SVZ/VZ = subventricular zone/ventricular zone; STR = striatum.

To study the role of ARX in IN development, *Arx* was ablated in INs by crossing *Arx^flox/+^; Rosa^Ai14/Ai14^* female mice with *Gad2Cre* homozygous males. *Gad2* is expressed in all IN lineage cells, beginning in the SVZ of MGE, lateral ganglionic eminence (LGE) and CGE on E10.^26^ The resulting *Gad2Cre-Arx* conditional knockout (cKO) mice (*Arx^flox/y^*; *Rosa^Ai14/+^*; *Gad2^Cre/+^*) die shortly after birth, similar to constitutive knockout (KO) mice.^23^ Although overall brain size and morphology appeared normal in cKO mice at E14.5 (Fig. 1A), the total number of tdTomato⁺ cells in the cortex was reduced (Fig. 1A–C and Supplementary Fig. 1), with a particularly pronounced decrease along the MZ migration path (Fig. 1A and D). There was an almost complete absence of cINs in the ventral-most region of the GE-cortical boundary—the initiation site for the cortical MZ migration stream (circled areas in Fig. 1A). Moreover, cINs migrated shorter distances compared with the WT (arrows indicating the migration front in Fig. 1B), with migrating cells tending to form abnormal clusters (asterisks in Fig. 1B). Consistent with the E14.5 findings, notably fewer cINs were detected in cKO mice at P0 (Fig. 1F), and they were disproportionately localized to deeper cortical layers, with few present in the upper layers (Fig. 1E and G).

To investigate the specific role of ARX in MGE-derived cINs, we used the *Nkx2.1CreER* line, which drives Cre expression in the MGE but not in the CGE or LGE^26^ (tamoxifen was administered at E12.5). These *Nkx2.1CreER-Arx* cKO mice exhibited IN phenotypes similar to *Gad2Cre-Arx* cKO mice, including a reduction of cINs in the MZ with relative sparing in the IZ/SVZ (Fig. 1H–K and Supplementary Fig. 2). Migrating cells formed abnormal clusters (asterisks in Fig. 1I) and migrated shorter distances when compared to WT (arrow in Fig. 1I). Many MGE-derived cINs aggregated around the developing striatum, failing to migrate to the cortex (arrowheads in Fig. 1H), compared to the broadly distributed cINs in WT. Similarly, by P0, a significant reduction of cINs, especially in the MZ, was observed, and most of the *Arx*-deficient *Nkx2.1*-lineage (tdTomato+) INs failed to migrate into the cortex and hippocampus (Fig. 1L–N). These findings were also observed in *Nkx2.1Cre*-driven cKO mice (Supplementary Fig. 2), which typically die before weaning. These data suggest that ARX enables entry of MGE-derived cells into the cortical MZ stream. Taken together, loss of ARX in the IN lineage leads to a marked reduction of cINs, particularly in the MZ, resulting in fewer upper-layer cINs and abnormal migratory behaviour.

### Single-cell RNA sequencing reveals pathways associated with cIN defects

**Figure 2:**
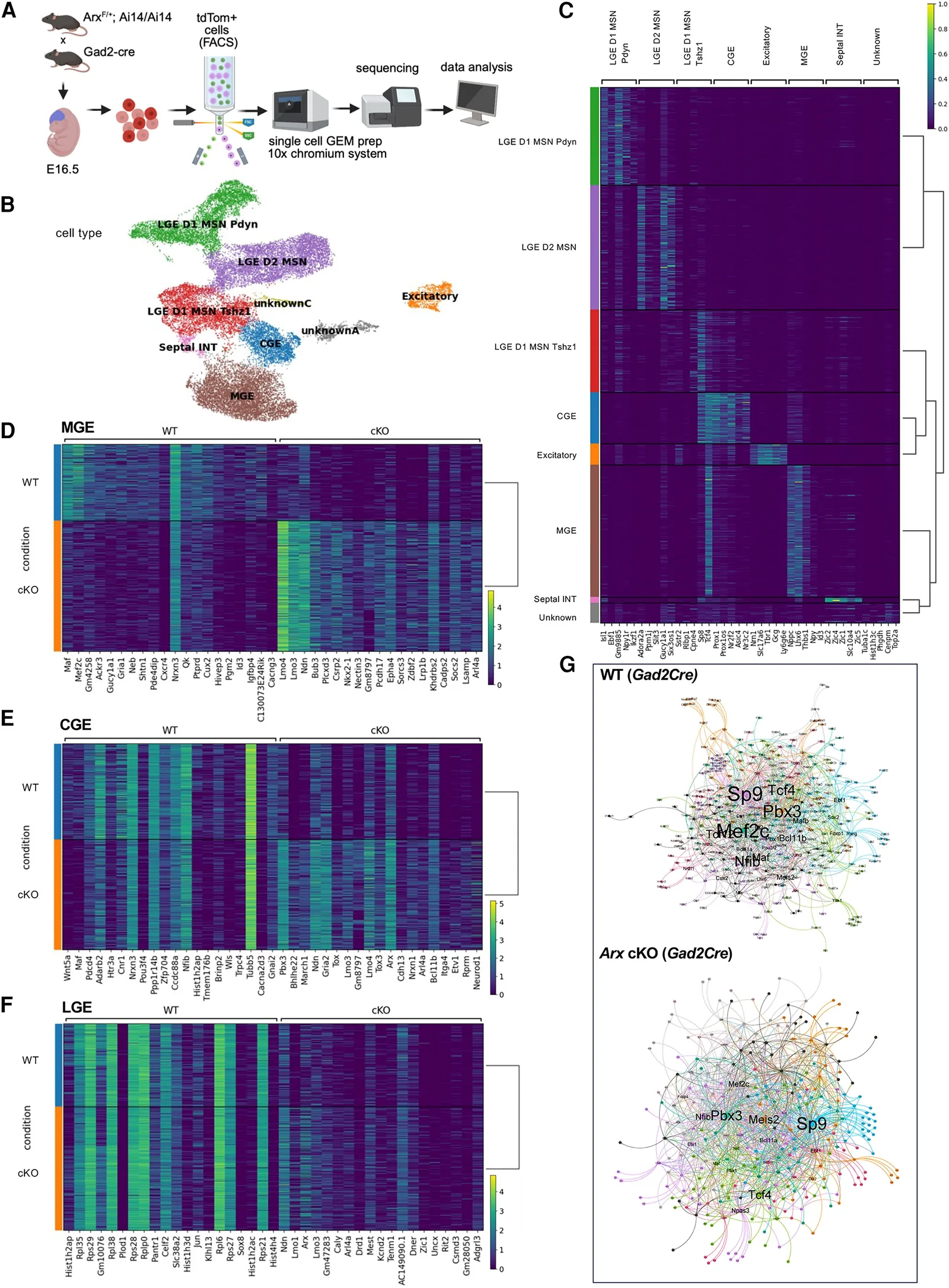
**Single cell transcriptome analysis of *Gad2Cre*- *Arx* conditional knockout and wild-type interneurons.** (**A**) Schematic diagram of overall experimental approach. The tdTomato+ interneurons (INs) were collected using FACS from embryonic Day (E)16.5 forebrains [*n* = 2 for wild-type (WT) and *n* = 3 for mutant (MT)]. Created in BioRender. Lim, Y. (2026) https://BioRender.com/rlu9vlv. (**B**) UMAP projection of INs identified eight clusters. (**C**) Heat map of cell clusters indicates differential expression of maker genes that best present IN subtypes. (**D**–**F**) Heat maps of MGE (**D**), CGE (**E**), LGE (**F**) clusters displaying differentially expressed genes in conditional knockout (cKO), compared to controls (WT). (**G**) Inferred transcription factor gene regulatory networks in WT and cKO MGE clusters using single-cell RNA sequencing data and chromatin accessibility data with CellOracle. CGE = caudal ganglionic eminence; FACS = fluorescence-activated cell sorting; LGE = lateral ganglionic eminence; MGE = medial ganglionic eminence; UMAP = Uniform Manifold Approximation and Projection.

To understand how changes in gene expression contribute to cIN phenotypes, we conducted single-cell RNA sequencing (scRNA-seq) using embryonic mice (E16.5). tdTomato^+^  *Gad2*-lineage cells were isolated from WT and cKO brains via fluorescence-activated cell sorting (FACS) and processed for scRNA-seq (Fig. 2A). After quality control, removing doublets, and normalization of sequencing results, we obtained a total of 27 585 high-quality cells from WT (11 224) and cKO (16 361) brains. The scRNA-seq analyses by scvi-tools^43^ identified several distinct clusters of cells, MGE, CGE, LGE, septal and excitatory neuron clusters (Fig. 2B and C).^19,44^ Three LGE clusters were further identified along with two ‘unknown’ clusters (Fig. 2B).^19,44^ The identified excitatory neuron cluster (NEUROG2⁺) contained tdTomato transcripts but lacked *Gad2* expression (Fig. 2B and Supplementary Fig. 3), potentially reflecting contamination or downregulation of *Gad2*.

Comparative scRNA-seq analysis of WT and *Arx*-deficient cells revealed transcriptomic changes across all GE cell clusters (Fig. 2D–F and Supplementary Fig. 3). The MGE and CGE clusters have more up- or down-regulated genes compared to the LGE (|FC| > 2; *P* < 0.05) (Supplementary Fig. 3E and Supplementary Table 1), possibly reflecting the higher expression of *Arx* in these two areas (Supplementary Fig. 3F). Highlighting the top 20 up- and down-regulated genes revealed cell cycle regulation (e.g. *Bub3*, *Csrp2*, *Ndn*)-,^45,46^ subtype differentiation (e.g. *Maf*, *Mef2c*)-^47-49^ and cell migration-associated genes (e.g. *Ackr3*, *Cxcr4*, *Epha4, ErbB4, Nrg1*, *Plxn3*)^50-52^ (Fig. 2D and E and Supplementary Fig. 3B and C). Interestingly, *Lmo1* was upregulated in all three GEs, and a few transcripts implicated in human conditions such as epilepsy, autism spectrum disorder, attention deficit, and Prader-Willi syndrome (e.g. *Ndn*, *Cadps2*, *Sortcs3*, *Lsamp*, *Nrxn*, *Cacng3*) were also detected (Supplementary Fig. 3). Consistent with the differentially expressed gene (DEG) analysis, the GSEA identified pathways involved in epithelial-mesenchymal transition (11.9%), axon guidance (10.1%) and EphrinB-EPHB pathway (5.5%), all of which are frequently associated with cell migration (Supplementary Fig. 3 and Supplementary Table 2).^21^ Next, gene regulatory network analyses performed using CellOracle^34^ demonstrated that major hub genes identified in WT samples, such as *Sp9*, *Maf*, *Mef2c* and *Pbx*, are no longer detected as major hubs (e.g. *Maf*) or show altered influence within the network (Fig. 2G). Together, these findings support perturbations in gene regulatory networks and the dysregulation of genes and pathways linked to cell cycle regulation, cell type specification and cell migration in *Arx* cKO mice.

### Cell type changes in *Arx*-deficient cell populations

**Figure 3:**
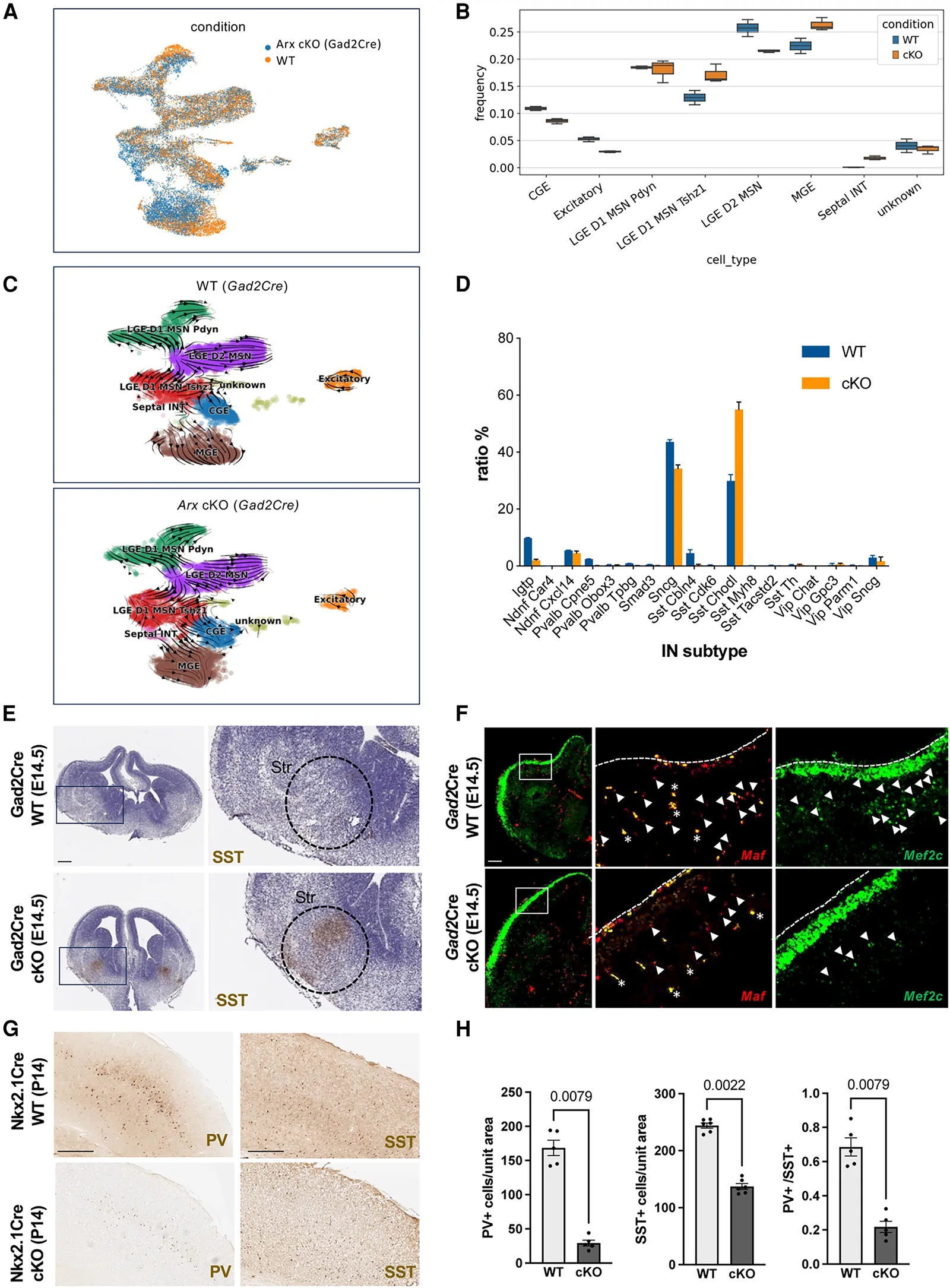
**Cell type changes in *Arx* conditional knockout mice.** (**A**) UMAP projection of identified cell clusters from *Gad2Cre-Arx* conditional knockout (cKO; blue) overlaid on wild-type (WT; orange) on embryonic Day (E)16.5. (**B**) Composition analysis of clusters. The MGE cell population is increased in *Gad2Cre-Arx* cKOs, but the CGE population is decreased. (**C**) RNA velocity analysis inferring differentiation trajectories. The overlaid arrows indicate the inferred developmental trajectory. (**D**) Single R analysis results using E16.5 single-cell RNA sequencing (scRNA-seq) data from Fig. 2 projected onto the adult scRNA-seq public data.^53^ (**E**) Representative images of somatostatin (SST) immunolabelling (DAB) of E14.5 coronal sections from WT or cKO mice. The dotted circles indicate the areas where SST expression is increased in cKOs. (**F**) Representative images of an RNAscope assay with *Maf* (red) or *Mef2c* (green) probes on E14.5 coronal sections from WT or *Gad2Cre-Arx* cKO mice. (**G**) Representative images of parvalbumin (PV) or SST immunolabelling of postnatal Day (P)14 coronal sections from WT or *Nkx2.1Cre-Arx* cKOs. The dotted circles indicate the areas where SST expression is increased in cKOs. (**H**) Quantification of PV+ or SST+ IN density and the ratio of PV INs to SST INs from P14 *Nkx2.1Cre*-*Arx* cKO or WT mice. *n* = 5 (PV) or 6 (SST) brains per genotype. Mann-Whitney test (two-tailed). *P*-values are shown. Scale bars = 200 µm. CGE = caudal ganglionic eminence; IN = interneuron; MGE = medial ganglionic eminence; UMAP = Uniform Manifold Approximation and Projection.

To compare cell clusters between *Gad2Cre*-*Arx* cKO and WT mice, the two single-cell datasets were aligned (Fig. 3A), and a composition analysis was performed (Fig. 3B). The mutant samples showed a reduction of CGE-clusters and an increase of MGE-clusters, suggesting a possible CGE-to-MGE identity shift. Subtype changes in the LGE clusters were also detected, with a decrease in D2 MSN, an increase in D1 MSN Tshz and no detectable changes within the D1 MSN Pdyn cluster (Fig. 3B). To gain further insights into this cell differentiation, we performed an RNA velocity analysis.^54^ Compared to clear, well-defined differentiation pseudo-time flow mapped in the WT MGE cluster (Fig. 3C), a broken MGE trajectory in the cKO indicated an impaired or disrupted differentiation process.^54^ Furthermore, while the WT cells exhibited distinct differentiation patterns between the MGE and CGE, without any connected trajectories, the cKO samples displayed a continued differentiation line without clear boundaries (consistent with network analysis in Supplementary Fig. 4A). Together, these findings suggested that the loss of *Arx* in the *Gad2*-lineage cells impacts their proper differentiation and provides evidence for an alteration of CGE identity to MGE.

Furthermore, a single R analysis that projected our E16.5 scRNA-seq data onto published adult data revealed IN subtype changes in cKO mice, highlighting an increased ratio of SST INs (SST chodl in Fig. 3D).^55^ Consistent with this, SST+ cells^11^ were dramatically increased in the ventral telencephalon of the cKO at E14.5 (circled areas in Fig. 3E), and early markers for SST INs were also increased (Supplementary Fig. 4B). We next performed immunolabelling for MAF and MEF2C, known to promote PV IN fate and strongly downregulated in the scRNA-seq analysis (Fig. 2D and E and Supplementary Fig. 3B and C).^47,48^ Both were reduced at E14.5 (Fig. 3F). As *Gad2Cre-Arx* cKO mice die shortly after birth, prior to PV expression, a reduction in PV could not be assessed. However, *Nkx2.1Cre-Arx* cKO mice that did survive longer showed a dramatic decrease in PV+ cINs (Fig. 3G and H). In these mice, a reduction in SST+ cINs was also observed at P14, although relatively spared compared with PV+ cINs (Fig. 3G and H). Taken together, these findings indicated that the loss of *Arx* in IN-lineage cells results in a disproportional loss of PV+ over SST+ cells.

### Cell cycle exit and cortical entry defects of *Arx*-deficient interneurons

**Figure 4:**
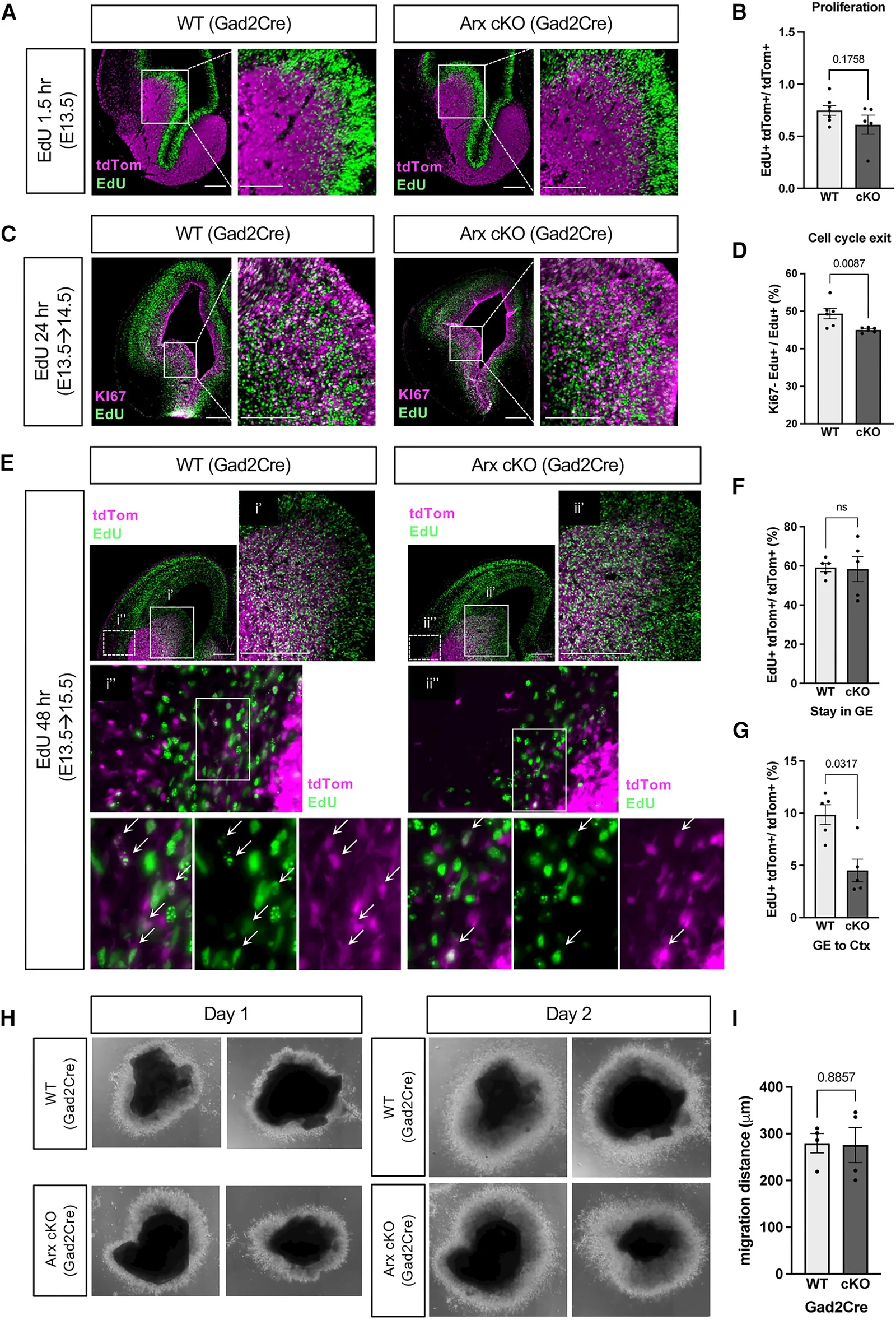
**ARX depletion leads to a disruption in the cell cycle exit and cortical entry of interneurons.** (**A**) Representative images of EdU (green) and tdTomato (magenta) immunolabelling on coronal sections of the wild-type (WT) and conditional knockout (cKO) (*Gad2Cre*) ganglionic eminences (GEs) with EdU injected at embryonic Day (E)13.5 and incubated for 1.5 h. *Right*: Magnified images from the boxed areas (*left*). (**B**) Quantification of the cell proliferation from the images taken as in **A**. *n* = 7 (WT) or 5 (cKO) mice. Mann-Whitney test (two-tailed). (**C**) Representative images of EdU (green) and Ki67 (red) immunolabelling on coronal sections of the WT and cKO (*Gad2Cre*) GEs with EdU injected at E13.5 and incubated for 24 h. *Right*: Magnified images from the boxed areas (*left*). (**D**) Quantification of the percentage of cells that exited the cell cycle. *n* = 6 brains per genotype. Mann-Whitney test (two-tailed). (**E**) Representative images of EdU (green) and tdTomato (red) immunolabelling on coronal sections of the WT (**i**) and cKO (**ii**) (*Gad2Cre*) GEs with EdU injected at E13.5 and incubated for 48 h. (**i′** and **ii′**) Magnified images of the lined boxed areas in **i** and **ii**; (**i′′** and **ii′′**) magnified images of the dotted boxed areas in **i** and **ii**; each of the three images below **i″** and **ii″** are magnifications of the boxed areas in **i″** and **ii″**; arrows in the bottom images indicate examples of cells double positive for EdU and tdTomato. (**F** and **G**) Quantification of the percentage of EdU+ cells among tdTomato+ cells entering the cortex (**F**) or staying in GE (**G**). *n* = 5 brains/genotype. Mann-Whitney test (two-tailed). (**H**) Two sets of the representative images of the explant cultures prepared at E13.5 and incubated for 1 (*left*) or 2 days (*right two*). (**I**) Quantification of migration distance measured from the explant culture images as shown in **H**. *n* = 4 brains/genotype (three cultures per brain). Mann-Whitney test (two-tailed). Error bars in each graph: mean ± standard error of the mean. Numbers over the bars = *P*-values. Scale bars = 200 µm.

To investigate the mechanisms underlying cIN defects following the loss of *Arx*, we examined multiple aspects of cIN development. Given that *Arx* expression begins in the SVZ of the GEs (Supplementary Fig. 3G), where intermediate progenitor cells proliferate and differentiate into neurons, we first assayed cell proliferation and cell cycle exit rates in the GEs. EdU labelling (injected at E13.5, incubated for 1.5 h) did not reveal a statistically significant change in the proportion of proliferating cells in *Arx* cKO (0.61 ± 0.09 for cKO and 0.75 ± 0.05 for WT; *P* = 0.1758) (Fig. 4A and B). However, the cell cycle exit rate, analysed by 24-h EdU labelling (injected at E13.5) in combination with Ki67 staining, revealed significant changes in *Arx* cKO (Fig. 4C and D). The percentage of cells exiting the cell cycle (EdU⁺ Ki67⁻/ EdU⁺) was lower in cKO GEs compared to WT (44.91 ± 0.14% and 49.5 ± 0.75%; *P* = 0.0087) (Fig. 4C and D). These findings indicated that more GE cells in cKO mice remained in the cell cycle, failing to exit and differentiate into neurons; this implied that the loss of cINs in cKO mice could, at least in part, be explained by a decreased cell cycle exit rate.

Given the extent of cIN loss observed in the *Arx* cKO mice (Fig. 1), the modest defect in cell cycle exit is unlikely to fully account for the loss, suggesting that additional mechanisms are involved. This prompted us to ask whether IN migration from GEs into the cortex was also affected. To address this, we conducted 48 h of EdU labelling (injection at E13.5) and found that significantly fewer EdU+tdTomato+ INs had crossed the boundary between the GE and cortex in cKO mice compared to WT (4.7 ± 0.96% and 9.9 ± 1.15%; *P* = 0.0317) (Fig. 4E and F). In contrast, the numbers of EdU+tdTomato+ INs located within the GE were comparable in both mice (57.38 ± 5.38% and 58.95 ± 2.53%; *P* = 0.7959) (Fig. 4G). These results indicated that ARX is necessary for normal entry into the cortex. To determine whether this was due to a primary defect in IN motility, a migration assay was performed using a GE explant.^39^ WT and cKO INs were found to migrate at similar rates and distances, suggesting that the failure of cortical entrance is not due to defects in IN motility (Fig. 4H and I). Taken together, our data indicate that the reduced number of cINs along the MZ path and, eventually, in the upper layer of the mature cortex results from a combination of defects, including the generation of cINs from progenitor cells and a failure of cINs to enter the cortical migration path.

### Abnormal regulation of *Lmo1* leads to defects in *Cxcr4*-mediated cortical entry of interneurons

**Figure 5:**
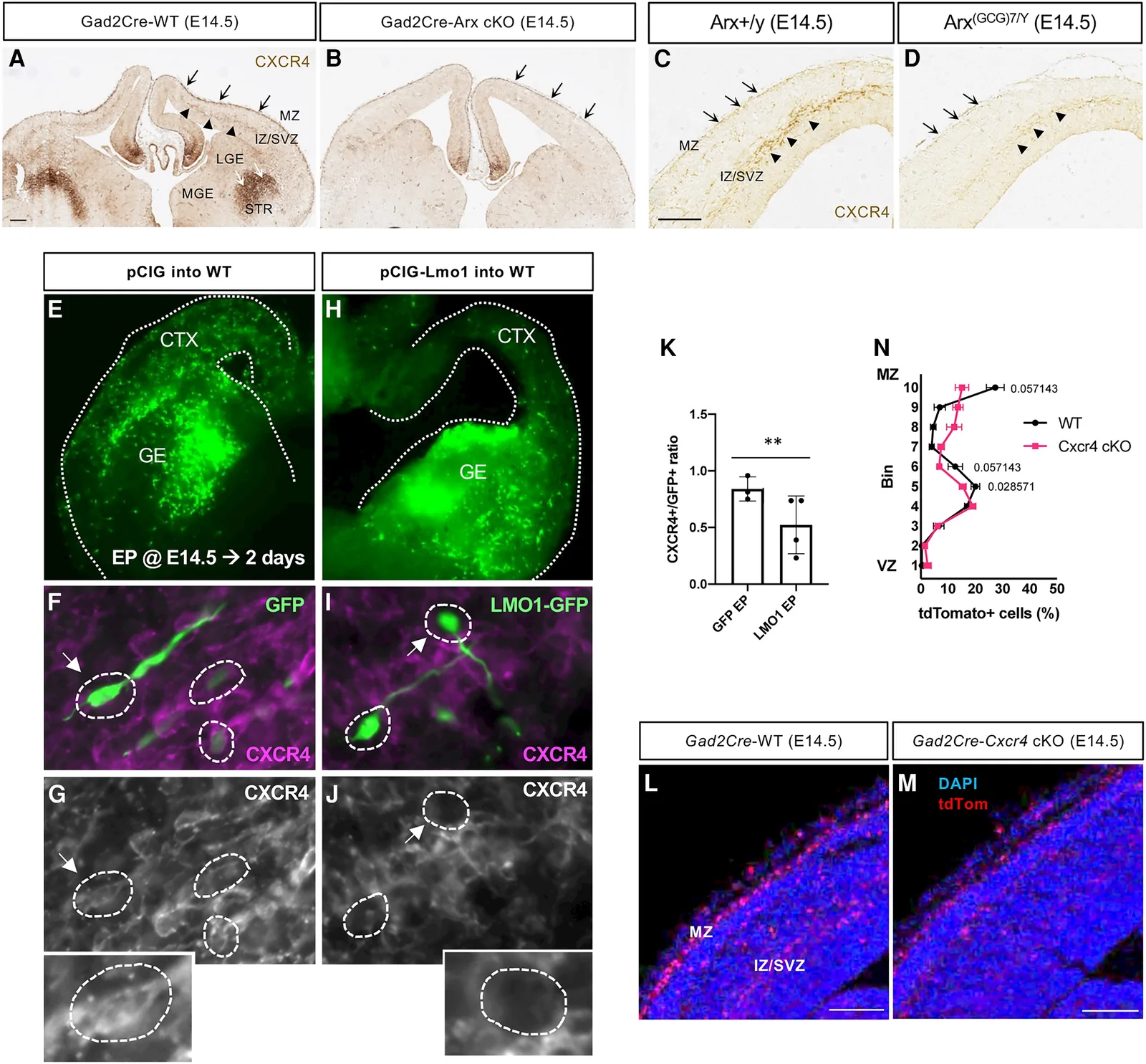
***Cxcr4* downregulation by *Lmo1* is in part responsible for cortical interneuron migration phenotype in *Arx* mutant mice**. (**A**–**D**) Representative images of CXCR4 immunolabelling (DAB) on coronal sections of wild-type (WT; **A** and **C**), *Arx* conditional knockout (cKO; **B**), or Arx^(GCG)7/y^ (**D**) brain. Arrows and arrowheads, CXC4+ cells in MZ and IZ/SVZ, respectively. (**E**–**J**) Forced expression of pCIG (**E**–**G**) or pCIG-Lmo1 (**H**–**J**) into the ganglionic eminence (GE) of WT brain slice cultures via electroporation at embryonic Day (E)14.5 (analysed 2 days later). Migrating interneurons (INs) electroporated with pCIG (green cells marked with dotted circles in **F**) expresses CXCR4 (**G**), but those with pCIG-*Lmo1* (green cells marked with dotted circles in **I**) lost CXCR4 expression (note no signal in dotted cells in **J**). (**K**) Quantification of the ratio of CXCR4+ cells among GFP+ cells in pCIG or pCIG-*Lmo1* electroporated slice culture. *n* = 3 (pCIG) or 4 (pCIG-Lmo1) cultures. Mann-Whitney test (two-tailed). **P < 0.01. Error bars: mean ± standard error of the mean (SEM). (**L** and **M**) Representative images of tdTomato immunolabelling on coronal sections of the WT (**L**) or *Cxcr4* cKO (*Gad2Cre*) (**M**). (**N**) Quantification of the percentage of the tdTomato+ cells in each bin to the total tdTomato+ cell numbers in all 10 bins in WT and cKO neocortices at E14.5. *n* = 4 mice (two sections per mouse was used). Error bars in each graph: mean ± SEM. Numbers next to the data points: *P*-values. Multiple Mann-Whitney tests. Scale bars = 200 µm. CTX = cortex; IZ/SVZ = intermediate zone/subventricular zone; MZ = marginal zone; VZ = ventricular zone.

To gain further insight into the potential mechanisms underlying the cIN MZ migration defect, we focused on one of the ARX downstream genes, *Cxcr4*, which encodes a receptor for the cortical guidance signal CXCL12 that attracts cINs towards the MZ.^51,52,56^  *Cxcr4* was selected because its loss is also accompanied by a reduction in MZ cIN migration and the number of upper-layer cINs.^51,52,56^  *Cxcr4* was also among the top 20 downregulated genes in the MGE clusters, based on scRNA-seq analysis (Supplementary Fig. 3). Its downregulation in the MZ and IZ/SVZ, as well as in the striatum, was confirmed by CXCR4 immunolabelling (Fig. 5A and B). Consistent with this, its expression was also reduced in *Arx^(GCG)7/Y^* mutant mice (Fig. 5C and D), which contain seven extra alanines in polyalanine tract 1, modelling one of the most frequent *ARX* variants in human patients.^24^ These mice also showed a mild reduction of DLX2+ INs in the MZ stream (Supplementary Fig. 5). These data suggest that the loss of CXCR4 potentially explains, at least in part, IN migration defects in *Arx* cKO mice. Unlike *Gad2Cre-Arx* cKO mice, *Arx^(GCG)7/Y^* mice survive into the late postnatal stage, allowing the assessment of PV+ and SST+ cINs. Both populations were reduced at P30 (Supplementary Fig. 6), consistent with a CXCR4-mediated defect in IN migration.

Curiously, the *Cxcr4* genomic locus was not detected in the ARX ChIP-seq analysis (Fig. 7), suggesting ARX might indirectly regulate it through another transcription factor. *Lmo1* was a likely candidate, given its consistent upregulation in multiple transcriptomic analyses of *Arx* mutant mice (Fig. 2D–F and Supplementary Figs 3B–D and 6B).^29,57,58^ More importantly, it has been shown to bind the *Cxcr4* genomic locus (Supplementary Fig. 8A).^59^ To test this hypothesis directly, we overexpressed *Lmo1* in the GE of WT brain slice cultures. Strikingly, most INs electroporated with *Lmo1* failed to migrate to the cortex (Fig. 5H), while those electroporated with GFP progressed normally (Fig. 5E). Consistent with these data, cells electroporated with *Lmo1* did not express *Cxcr4* (Fig. 5I–K), while those electroporated with GFP showed normal *Cxcr4* expression (Fig. 5F, G and K), supporting *LMO1*'s role as a direct transcriptional repressor of *Cxcr4*. These data suggested that upregulation of *Lmo1* in *Arx*-mutant INs, at least in part, is responsible for the IN migration/distribution abnormalities observed.

To further test this, we generated *Gad2Cre*-*Cxcr4* cKO lines crossed with the *Ai14* mouse line to abrogate *Cxcr4* in the *Gad2*-lineage INs, while simultaneously labelling *Cxcr4*-abrogated cells with tdTomato. *Cxcr4* cKO mice exhibited a cIN migration phenotype (Fig. 5L and M) similar to that observed in several *Arx* mutant mice (Fig. 1 and Supplementary Fig. 5), supporting the idea that downregulation of *Cxcr4* by upregulated *Lmo1* contributes to the cIN migration defect in *Arx* mutant mice. These findings revealed a novel mechanism by which ARX regulates cIN migration.

### Disordered cell-cell communication upon loss of *Arx*

**Figure 6:**
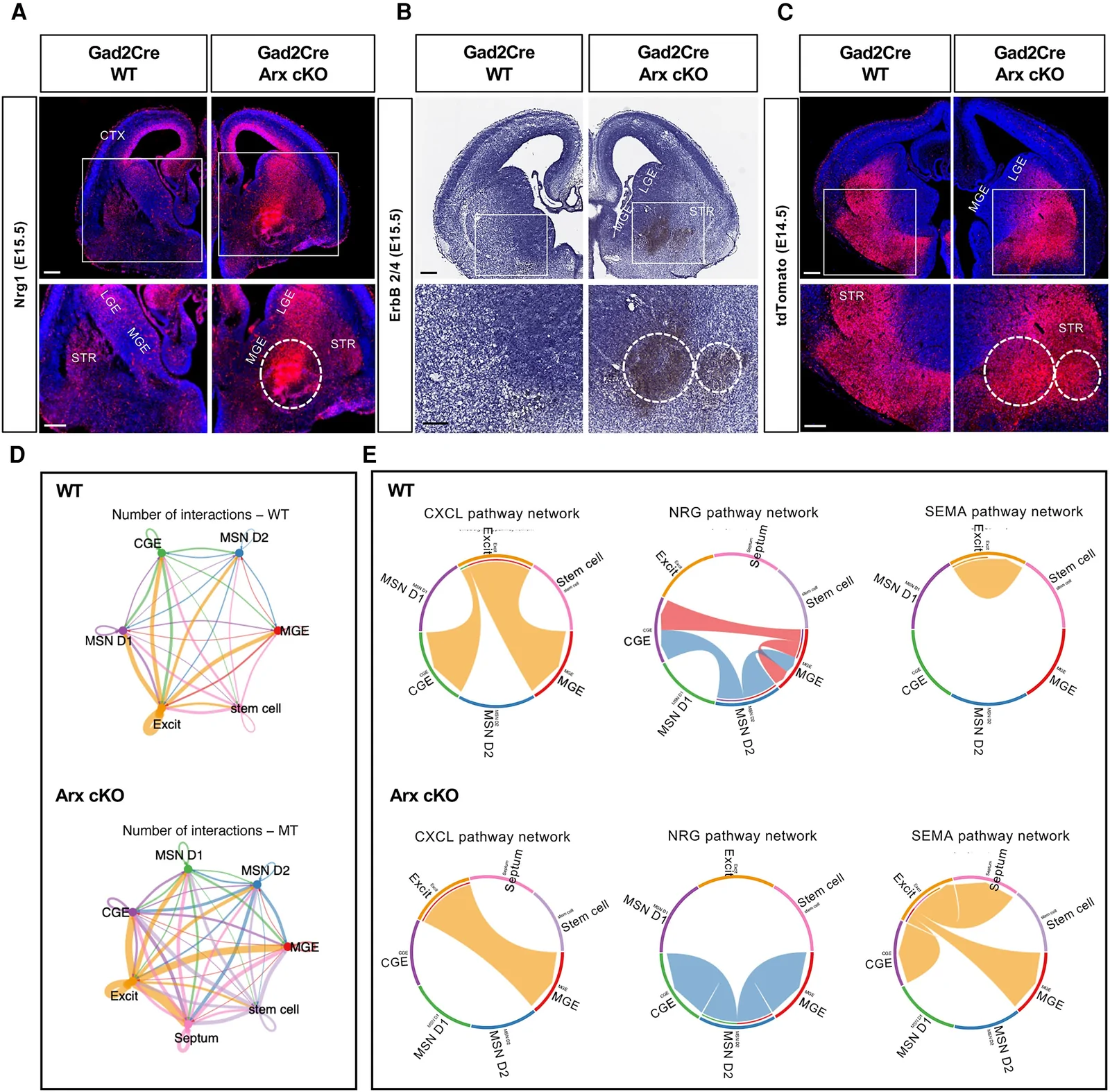
**Interneuron migration-associated ligands and receptors are mis-regulated in *Gad2Cre-Arx* conditional knockout mice.** (**A**) Representative images of *Nrg1* RNA *in situ* hybridization (RNAscope) on coronal sections of wild-type (WT; *left*) or *Arx* conditional knockout (cKO; *right*) brain. *Bottom*: Magnified images of boxed areas (*top*). Dotted circle: strong ectopic expression of *Nrg1*. (**B**) Representative images of ErbB2/4 immunolabelling (DAB) on coronal sections of WT (*left*) or *Arx* cKO (*right*) brain. *Bottom*: Magnified images of boxed areas (*top*). Dotted circles: strong ectopic expression of ErbB2/4. (**C**) Representative images of tdTomato immunolabelling on coronal sections of WT *(left*) or *Arx* cKO (*right*) brain. Dotted circles: clustered tdTomato+ cells in the areas matching with ectopic upregulation of *Nrg1* and ErbB2/4 (compare dotted circles in **A** and **B**). These sections are the same sections used in Fig. 1A. (**D**) Circle plots demonstrating the numbers and strengths of interactions between each cell type identified in single-cell transcriptomics of WT and *Arx* cKO (mutant, MT). The line represents the connection between two cell types; the line thickness represents the interaction strength; the loop represents the interaction within the same cell type. (**E**) Chord diagram depicting all the significant ligand-receptor pairs (cell-cell interactions) between different cell types in WT and cKO. Scale bars = 200 µm. CTX = cortex; IZ/SVZ = intermediate zone/subventricular zone; LGE = lateral ganglionic eminence; MGE = medial ganglionic eminence; MZ = marginal zone; STR = striatum.

The cIN migration defects in *Arx* cKO mice are more severe than those observed in *Cxcr4* cKO mice (Figs 1A and 5L and M), suggesting that additional factors, beyond *Cxcr4*, might be disrupted in *Arx* cKOs. Neuregulin1 (*Nrg1*) and its receptor *ErbB4,* known to influence cIN migration,^22,60^ are upregulated in the MGE and CGE, as shown by scRNA-seq analyses (Supplementary Fig. 7) and immunolabelling (Fig. 6A and B). Normally, *ErbB4*-expressing INs traverse the LGE that is NRG1+ but SEMA3A/3F-. The ectopic upregulation of *Nrg1* and *ErbB4* in the MGE and CGE raises the possibility that INs are misguided to the MGE and CGE. Indeed, tdTomato+ *Gad2*-lineage INs were found to accumulate adjacent to the striatum (dotted circles in Fig. 6C), the location of ectopic *Nrg1* and *ErbB4* expression (dotted circles in Fig. 6A and B). Furthermore, semaphorin 3A (SEMA3A) and its receptor neuropilin 1 (NRP1), which normally form a striatal repellent signal, are upregulated in cKOs (Supplementary Fig. 7A). This likely results in a cell-cell communication abnormality. Together, these data provided a model in which the loss of Cxcr4 and the upregulation of *Nrg1*-*ErbB4* and *Sema3a*-*Nrp1* contribute to the failure of INs to enter the cortex.

CellChat, which provides quantitative inference and analyses of cell-cell communication networks from scRNA-seq data, was used to evaluate significant ligand-receptor expression changes.^33,61^ The number and strength of cell-cell interactions in communication networks were predicted to be higher in cKO than WT brains (Fig. 6D), suggesting disrupted regulation and chaotic interactions among cKO INs. The changes in the CXCL, NRG and SEMA signalling pathways are displayed in Chord diagrams that show significant interactions between different cell types; again, more cell-cell interactions in cKO than WT brains (Fig. 6E). Additionally, CellChat analysis enabled the prediction of key incoming and outgoing signals for specific cell types by quantitatively measuring ligand-receptor networks with pattern recognition approaches. As shown in the heat map of multiple signal strengths (Supplementary Fig. 7B), comparison of both outgoing and incoming signalling patterns highlighted the alterations in the ligand-receptor interaction landscape in each cell type upon *Arx* deletion (Supplementary Fig. 7B). Taken together, these analyses provided further evidence that the loss of *Arx* disrupts cell-cell communications, resulting in IN migration defects.

### Identification of ARX direct target genes for interneuron differentiation and migration

**Figure 7:**
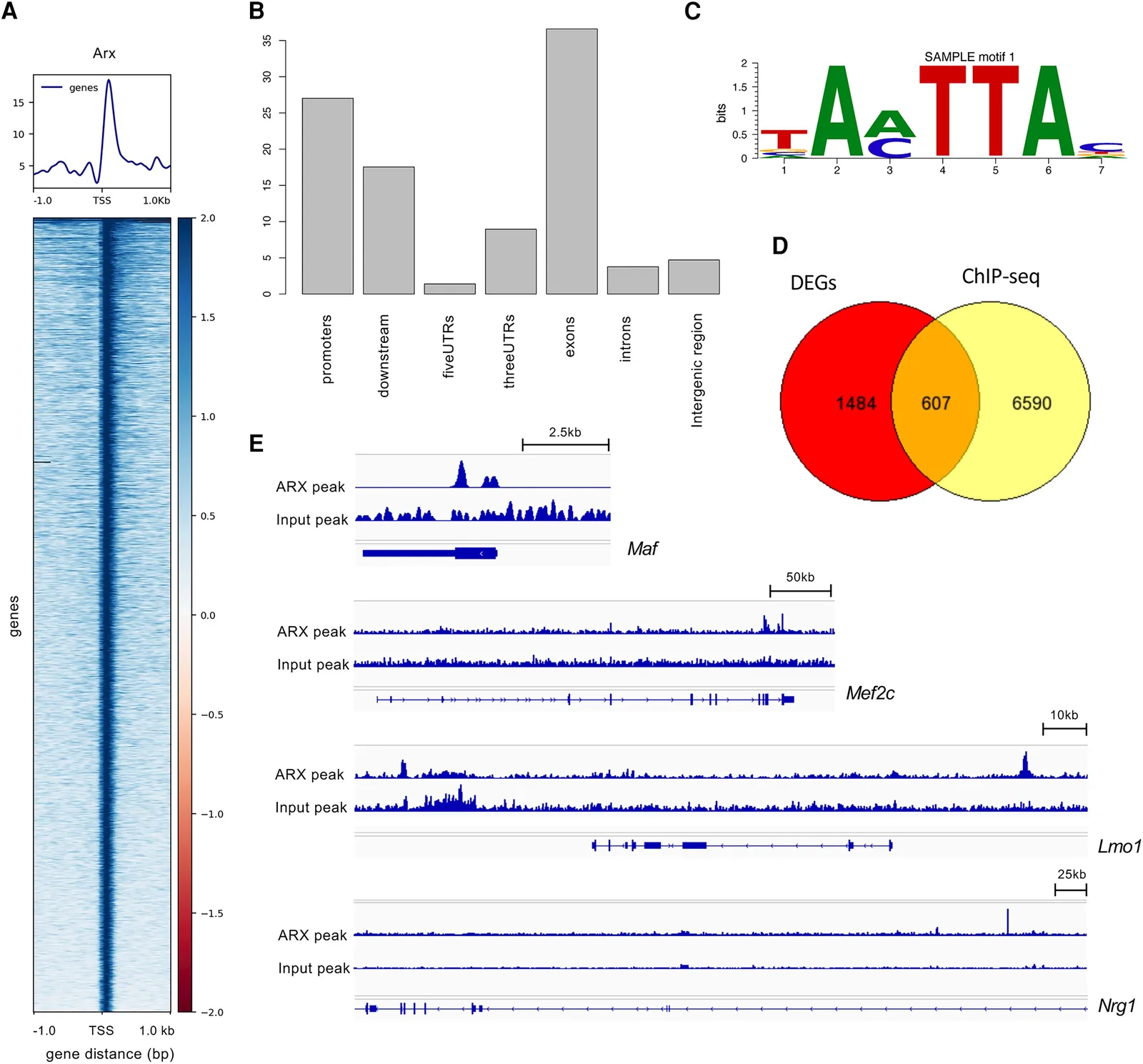
**ARX ChiP sequencing analysis.** (**A**) Heat map of ARX binding sites near transcription start site (TSS). (**B**) ARX binding regions of the mouse genome. (**C**) ARX binding site sequences in ChIPed DNA. (**D**) Comparison of differentially expressed genes (DEGs) identified by single-cell RNA sequencing (scRNA-seq) versus the genes identified by ARX ChIP sequencing (ChIP-seq). (**E**) Examples of gene loci (*Maf*, *Mef2c*, *Lmo1* and *Nrg1*) identified as ARX binding sites in ARX ChIP-seq analysis. ChIP = chromatin immunoprecipitation.

To identify ARX direct target genes, ChIP-seq assays were performed using E14.5 mouse forebrains (Fig. 7 and Supplementary Table 3). Binding site analyses revealed that ARX binding sites are located near the transcription start site (TSS) and are enriched in promoters and exons (Fig. 7A and B). We also identified enriched consensus binding motifs for ARX (Fig. 7C) and found that 607 of the 7197 genes identified by ChIP-seq were among the 2091 DEGs (Fig. 7D). The ARX ChIP-seq data confirmed that ARX binds to genomic loci of the key DEGs identified from the scRNA-seq analysis (Supplementary Fig. 3), including (i) *Bub3* and *Csrp2*; (ii) *Maf* and *Mef2c*, (iii) *Lmo1*; and (iv) *Nrg1* and *ErbB4* (Supplementary Fig. 8B). *Bub3* and *Csrp2*, upregulated in the MGE clusters, are known to function in cell cycle regulation and in the maintenance of stemness.^45,46^  *Maf* and *Mef2c*, downregulated genes in the MGE and CGE clusters, direct PV IN subtype specification at the expense of SST INs (Fig. 7E).^47-49^  *Lmo1*, upregulated in the MGE, CGE and LGE clusters, as well as *Nrg1* and *ErbB4*, regulates IN migration (Figs 5 and 7E and Supplementary Fig. 8B).^22,60,62^ Taken together, these data provide further evidence that ARX plays a role in multiple aspects of IN development by regulating the transcription of a subset of genes involved in the cell cycle, subtype specification and migration.

Additionally, *Nkx2.1* was identified as a direct transcriptional target of ARX (Supplementary Fig. 9A). ARX binding sites in *Nkx2.1* overlap with those of DLX2 binding,^63^ providing evidence for their regulatory function in *Nkx2.1* transcription. It is worth noting that we recently demonstrated physical interaction between ARX and DLX2.^64^ During normal development, *Nkx2.1* is predominantly expressed in MGE progenitor cells, downregulated as the cells begin to migrate and differentiate, and not detected in the LGE- or CGE-lineages, or within the cortex (Supplementary Fig. 9B–D). However, in *Arx* cKO brains driven by *Gad2Cre* or *Nkx2.1Cre*, ectopic NKX2.1*+* cells were detected in the cortex and CGE (Supplementary Fig. 9B–D), and NKX2.1 expression persisted in *Arx*-deficient cINs (tdTomato+) at P0, while no WT cINs (tdTomato+) expressed NKX2.1 (Supplementary Fig. 9E and F). These ectopically located NKX2.1+ cells appear to be MGE-derived, given that they are detected both in the *Nkx2.1Cre*- and *Gad2Cre*-*Arx* cKO brains. Our data suggest that during normal development, ARX represses *Nkx2.1* as MGE-derived cIN precursors begin to migrate out of the ventricular zone and differentiate.

### Cortical interneuron loss in a patient carrying a pathological variant of *ARX*

**Figure 8:**
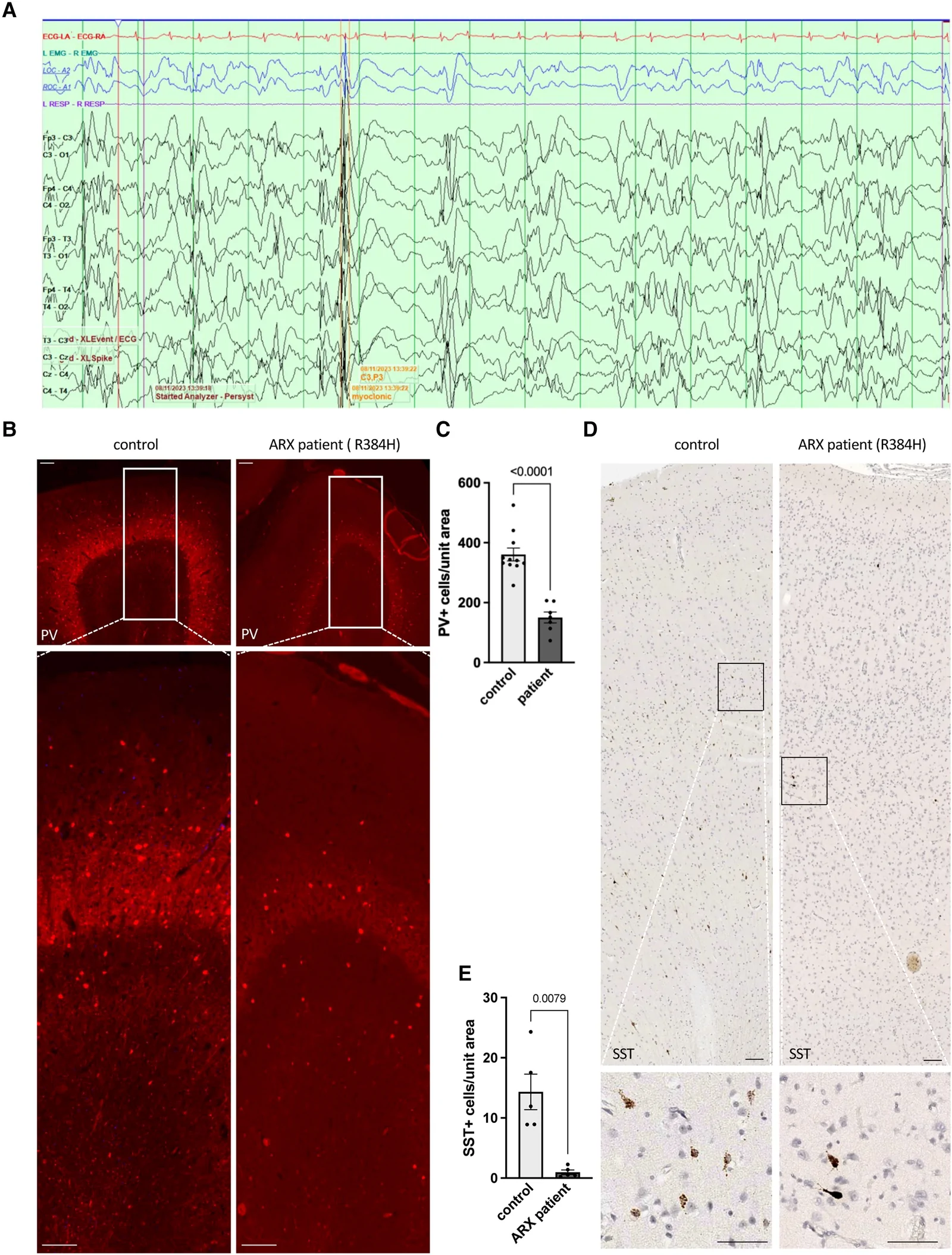
**Cortical interneuron (cIN) defects in the developmental epilepsy patient with *ARX* pathogenic variant** (**R384H**). (**A**) EEG data of the patient at 15 months old indicates a myoclonic seizure. (**B** and **D**) Representative images of parvalbumin (PV) immunofluorescence labelling (**B**) and somatostatin (SST) RNAscope (**D**) on post-mortem cortex (occipital and prefrontal, respectively) from the same patient with *ARX* variant (at 22 months old) and an age-matched control. *Bottom*: Magnified images of the boxed areas in the *top*. (**C** and **E**) Quantification of the PV+ (**C**) and SST+ (**E**) cell density (cell numbers/10 mm^2^) measured from the post-mortem cortex of the patient (*n* = 7 regions of interest for PV; *n* = 4 for SST) and control (*n* = 11 regions of interest for PV; *n* = 7 for SST). Number over the bar: *P*-value. Mann-Whitney test. Error bars: mean ± standard error of the mean. Scale bars = 100 µm.

*ARX* variants in humans cause a wide spectrum of disorders from X-linked lissencephaly with abnormal genitalia (XLAG) to X-linked infantile spasms syndrome (ISSX) without accompanying brain malformations.^65^ The severe phenotypes are associated with deletions, truncations or alterations in conserved homeodomain amino acids, whereas epilepsy and intellectual disabilities without brain malformations are linked to polyalanine expansions or single amino acid changes outside the homeodomain.^4^ We obtained the post-mortem brain of a 22-month-old male with intractable symptomatic multifocal epilepsy carrying a pathogenic hemizygous variant of *ARX* (p. Arg384His; c.1151G>A in exon 4; clinical information is provided in the ‘Materials and methods’ section). An EEG at 15 months of age demonstrated high voltage multi-focal interictal spikes with high-voltage disorganized delta, consistent with severe epileptic encephalopathy (Fig. 8A). A high-voltage polyspike followed by an electrodecrement is shown that is time-locked with a myoclonic seizure (Fig. 8A). Immunofluorescence labelling and RNAscope analyses revealed prominent cIN defects in the post-mortem brain of the same patient, compared with the age-matched control brain; significant losses of both PV+ and SST+ INs were observed (Fig. 8B–D), substantiating our data in *Arx* mutant mice.

## Discussion

ARX is a key transcription factor for cIN development and is involved in a spectrum of neurologic disorders.^66^ Elucidating the role of ARX and its associated transcriptional networks is essential to better understand these conditions. Our findings provide evidence that ARX regulates multiple aspects of cIN development by directly or indirectly modulating target gene expression.

Our cell cycle assays showed that ARX plays a role in cell cycle exit in GE SVZ cells, and disruptions in this process appear to interrupt the transition from proliferation to differentiation. This is in accordance with abnormal upregulation of *Csrp2*, known to promote stemness,^46^ and *Bub3*, a cell cycle checkpoint protein often overexpressed in tumour cells.^45^ These two genes are direct transcriptional targets of ARX (Supplementary Fig. 8), and their upregulation in *Arx*-deficient IN precursors likely keeps them in a proliferation status, suppressing differentiation. Thus, ARX seems to promote IN precursor differentiation by inhibiting cell cycle progression, a function also observed with other transcription factors.^67^ Interestingly, this is different from observations of cortical projection neuron precursor cells, where ARX normally represses *Kip2*, a cell cycle inhibitor, thus suppressing differentiation.^68^ The distinct functions of ARX in cortical versus GE precursor cells appear to be consistent with its differential expression patterns; in cortical (pallial) progenitors, *Arx* is primarily expressed in the VZ, whereas in GE (subpallial) progenitors, it is expressed in the SVZ and in differentiated INs.^69^

Our scRNA-seq data, combined with immunolabelling analyses, demonstrated that ARX also participates in IN subtype specification. *Arx* depletion from IN precursors leads to a significant shift from CGE to MGE identity. Additionally, the abnormal upregulation of *Sst* and downregulation of *Maf* and *Mef2c,* which are direct targets of ARX and early markers of PV INs,^48^ suggest cell-type changes. In fact, these changes in embryonic brains resulted in a reduced ratio of PV+ to SST+ INs in P14 cKO brains. This raises the possibility of a fate shift of PV+ cells to SST+ cells, similar to that observed in *Mafb* and *c-Maf* double cKO mice.^47^ Consistent with these data, the early PV IN markers (e.g. *Mef2c*, *Maf*, *Angp2*, *Crbp1*, *St18*) are decreased in cKO MGE clusters, while early SST IN markers (e.g. *Sst*, *Tspan7*, *Cacna2d3*, *Nxph1*) are increased. Interestingly, these *Arx* cKO phenotypes resemble those of the *Sp9* KO mice.^70^ This resemblance is expected, given that *Arx* is directly regulated by SP9.^70^

Our data, suggesting a role for ARX in promoting CGE (over MGE) and PV (over SST) IN fates, add another layer of complexity to ARX and its associated transcriptional regulatory networks, especially in relation to previous studies with *Lhx6* mutant mice.^71^ It has been reported that LHX6 directly regulates *Arx* expression, and the loss of LHX6 results in partial re-specification of MGE to CGE.^71^ Furthermore, the transduction of ARX rescues this phenotype in *Lhx6* mutant mice. Although our data, suggesting a role for ARX in promoting CGE fate, appear to contradict their conclusions on ARX function (promoting MGE fate), our results supporting ARX's role in promoting PV fate agree with their observation of a higher rescue rate in PV INs over SST INs by *Arx* transduction. We speculate that the role of ARX in IN fate specification may be more complicated than simply promoting MGE or CGE fate, requiring an understanding of the whole gene regulatory network.

The significant loss of *Arx*-deficient cINs in the MZ stream provides us with a unique opportunity to study the biology of IN migration. For example, *Nkx2.1* is a direct transcriptional target of ARX (Supplementary Fig. 8) and one of the top 20 upregulated genes in the MGE cluster. Considering downregulation of *Nkx2.1* in postmitotic cINs is necessary for them to enter cortical migration streams,^72^ we speculate that the observed IN migration defects in *Arx* cKO mice (as well as in null KO mice)^23^ may be attributed, in part, to a failure in downregulating *Nkx2.1*.

Another direct target of ARX involved in cIN migration is *Lmo1*, which is upregulated in *Arx* KO, cKO and *Arx^(GCG)^*^7^ mice. Interestingly, LMO1 directly represses *Cxcr4* expression, as indicated by previously reported ChIP-seq and our slice culture electroporation data.^59^ Considering its role in INs as a chemokine receptor for CXCL12, which attracts INs to the MZ and SVZ/IZ of the cortex,^73,74^ the loss of CXCR4 certainly plays a role in the observed migration defect. In addition, alterations in guidance signals (e.g. ectopic upregulation of *Nrg1* and its receptor *ErbB4*) as well as repulsive signals (e.g. upregulation of *Sema3* and its receptor *Nrp1*) appear to create a chaotic environment that inhibits cIN entry into the cortex. Future studies will assess the roles of Nrg1/ErbB4 and Sema3/Nrp1 in directing cINs out of the GE and into the cortex, in the context of ARX function.

We note that previous studies reported no significant changes in cIN numbers or distribution in *Arx* polyalanine expansion mouse models, in contrast to our data.^75^ We surmise this is due to differences in methodologies; the use of 3D image analysis of tissue-cleared, immunolabelled brains permits the counting of all labelled neurons rather than subsets, ostensibly a more sensitive measure.

Finally, the reduced number of PV+, as well as SST+, INs in the cortex of the patient with infantile spasms carrying a pathogenic *ARX* variant (R384H) demonstrates that a single amino acid change in ARX is associated with cIN defects like those seen in our mutant mouse models. We note that the severity of the SST IN loss in the patient is worse than that observed in mice. Although perinatal death of the *Gad2Cre*-Arx cKO (P0) and *Nkx2.1Cre*- *Arx* cKO (P0–P14) hinders seizure characterization, these findings in the human brain support the hypothesis that abnormal cIN development and migration play a role in the pathogenesis of seizure phenotypes in patients with this variant.

In summary, we have provided evidence that ARX is a hub transcription factor with key roles in the generation, fate specification and migration of cINs through the regulation of gene transcription networks during brain development. ARX controls multiple steps in IN differentiation by directly or indirectly regulating complex GRNs. Together, our findings provide novel insights into ARX’s role in cIN development and how this role is disrupted in disease.

## Supplementary Material

awag036_Supplementary_Data

## Data Availability

Data supporting the findings of this study are available from the corresponding author upon reasonable request. The accession numbers for the GEO dataset we uploaded are GSE273676, GSE274243 and GSE274244.
