# Supplementary material for: ARX mutation-associated interneuron defects provide insights into mechanisms underlying developmental epilepsies
Source: Brain. 2026 Feb 3;149(9):3197–213. doi: 10.1093/brain/awag036 (PMC13548863; doi:10.1093/brain/awag036)
Supplement: Supplementary file 1 [file awag036_supplementary_data.zip › brain-2025-02544-File010.pdf]

Supplementary Figure 1

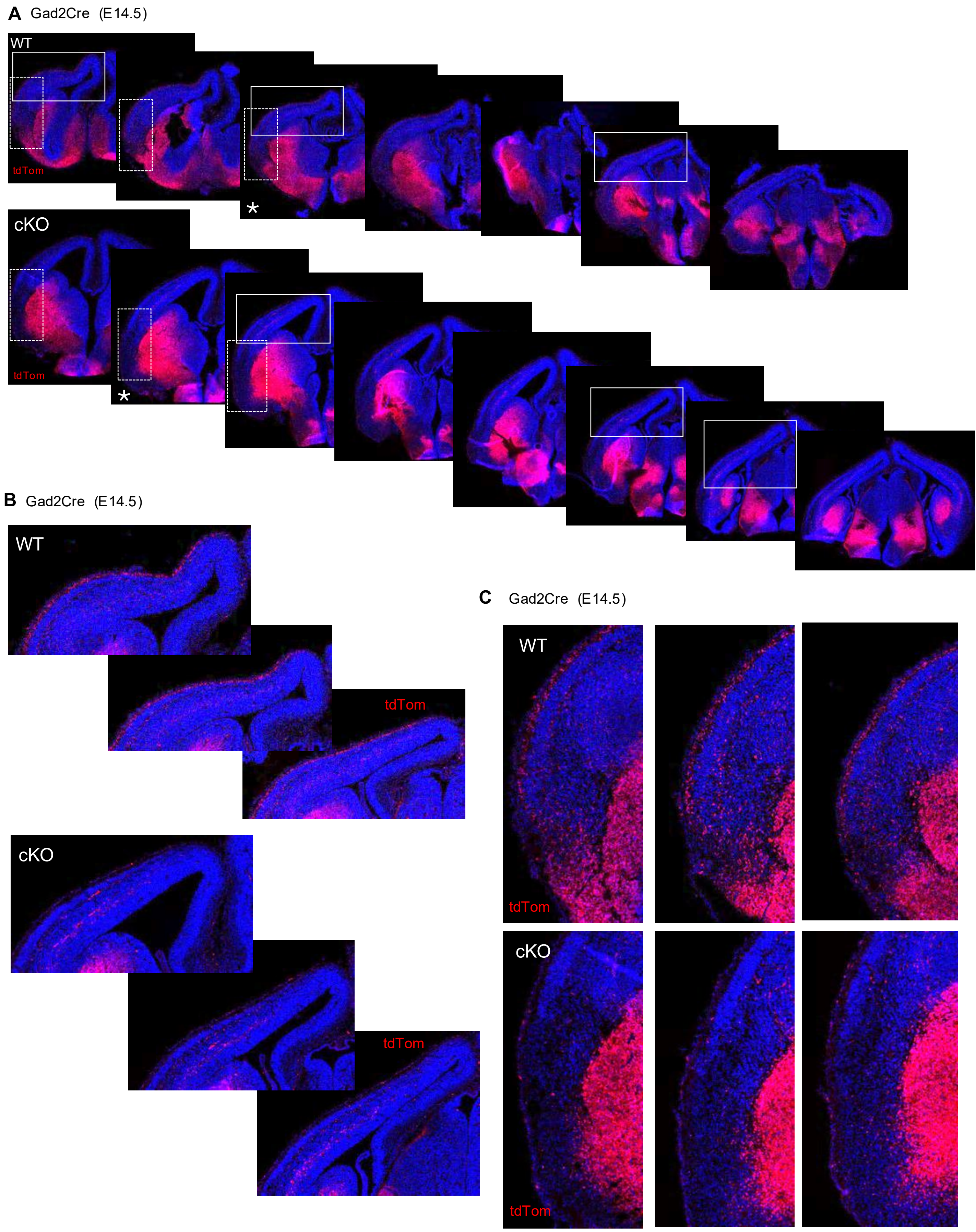

Supplementary Fig 1. **(A)** A representative series of coronal sections from *Gad2Cre-Arx* cKO or WT brains (E14.5) immunolabeled with tdTomato (red) and nuclear stained with DAPI (blue). Asterisks indicate the same sections shown in Fig. 1A. (**B)** Magnified images of solid boxed areas in A. (**B**) Magnified images of dotted boxed areas in A.

Supplementary Figure 2

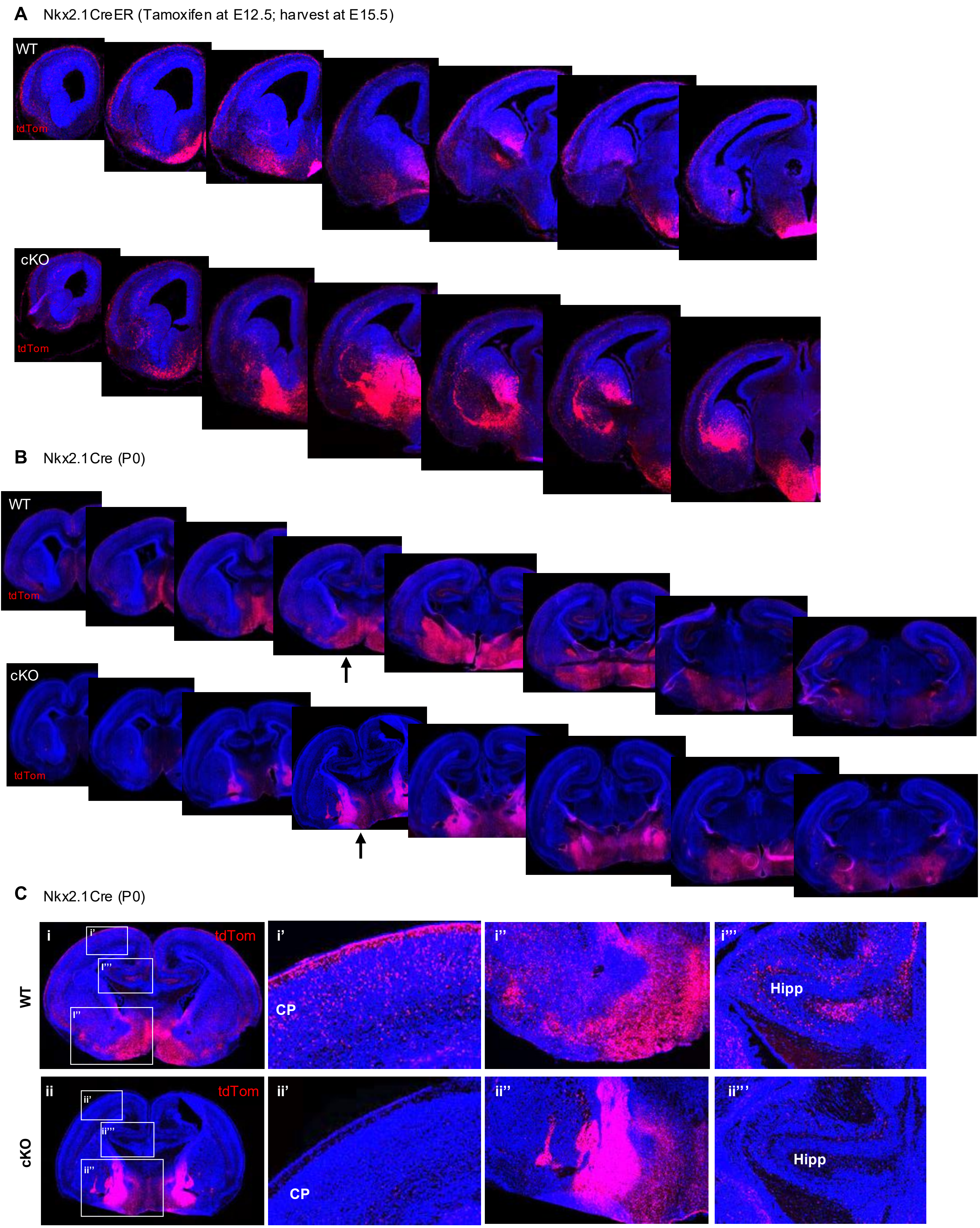

Supplementary Fig 2. (**A**) A representative series of coronal sections from *Nkx2.1CreER-Arx* cKO or WT brains (E15.5) immunolabeled with tdTomato and nuclear stained with DAPI (blue). (**B**) A representative series of coronal sections from *Nkx2.1Cre-Arx* cKO or WT brains (P0) immunolabeled with tdTomato. (**C**) Representative images of Nkx2.1Cre-Arx cKO or WT (P0) brain sections marked with arrows in B. I', ii', and iii' are magnified images of boxed areas in i. CP, cortical plate; Hipp, hippocampus.

Supplementary Figure 3

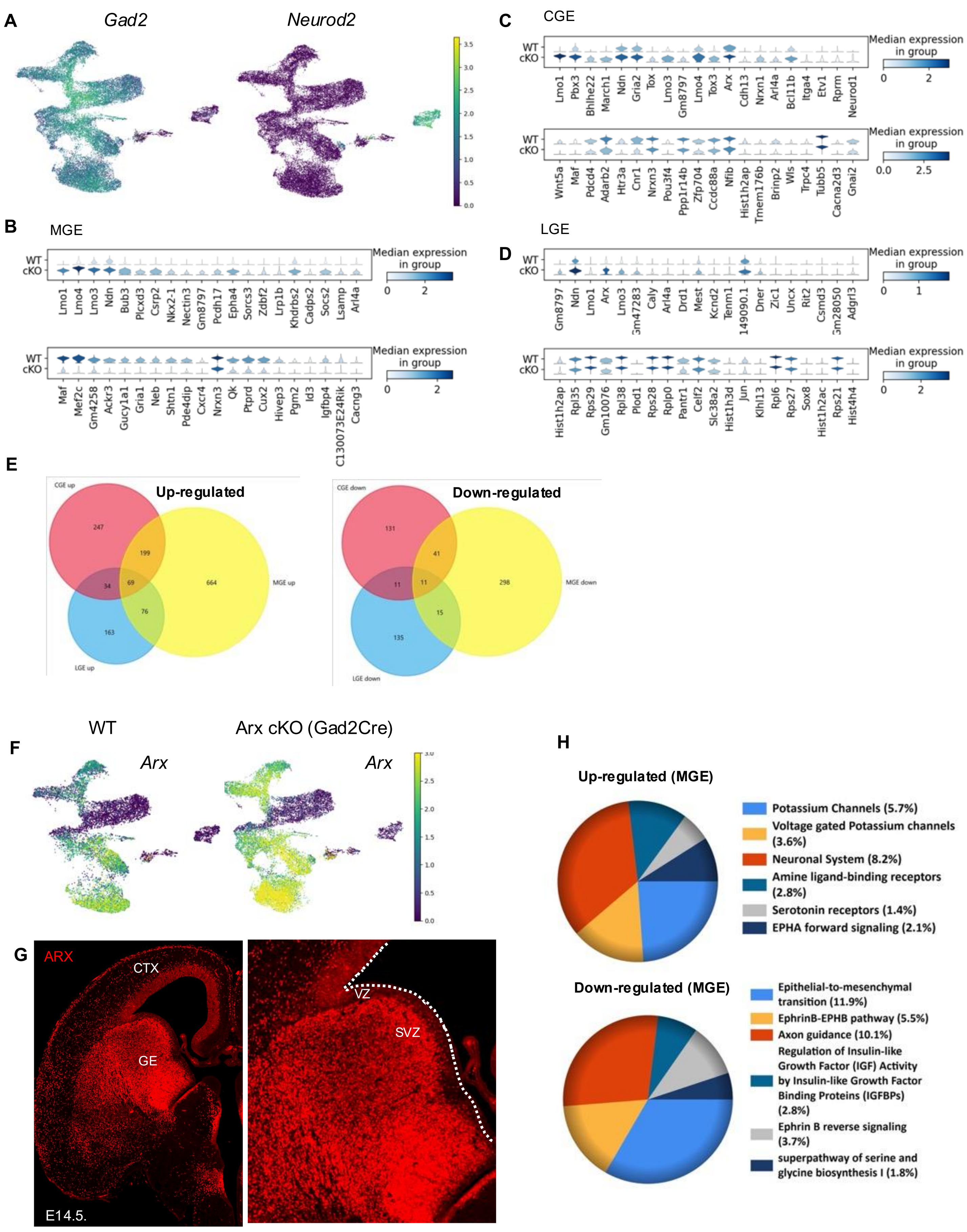

Supplementary Fig 3. (**A)** UMAP plot showing expression of *Gad2* and *Neurod2* transcripts in *Gad2* lineage cINs. (**B-D**) Top 20 up (top)- and down-regulated (bottom) genes from MGE (B), CGE (C), or LGE (D) clusters. (**E**) Venn diagram of up and downregulated genes identified from scRNA-seq in MGE, LGE and CGE INs (**F**) UMAP plot showing expression of *Arx* transcripts in *Gad2Cre-Arx* cKO and WT cell clusters. (**G**) Left: a representative image of a coronal section of the mouse brain (E14.5) immunolabeled with anti-ARX antibody. Right: a magnified image of the GE area from the image on the left. (**H**) Gene set enrichment analyses of the upregulated and downregulated genes in MGE INs. Abbreviation: CGE, caudal ganglionic eminence; LGE, lateral ganglionic eminence; MGE, medial ganglionic eminence.

Supplementary Figure 4

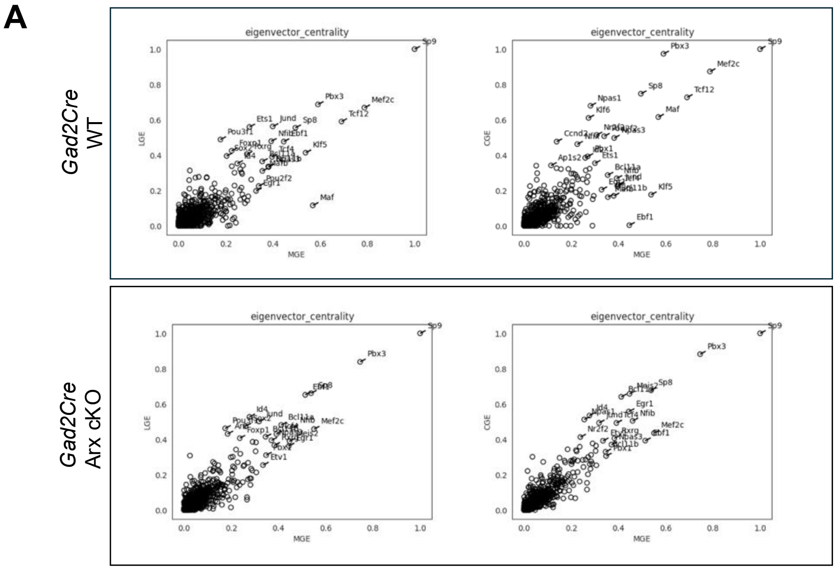

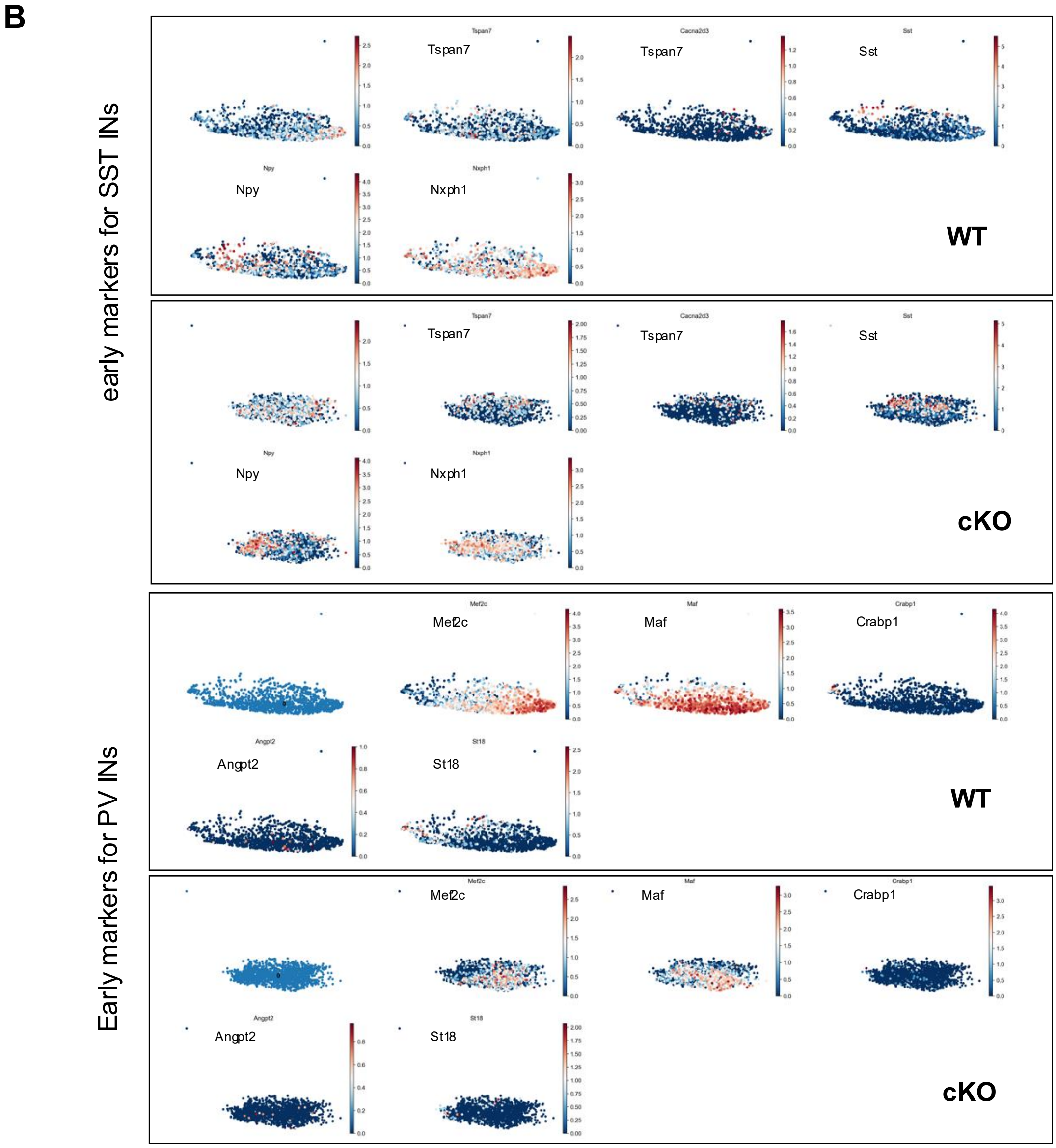

Supplementary Fig 4. (**A**) Eigenvector centrality plots obtained from gene regulatory network analysis of Gad2Cre-Arx cKO and WT MGE clusters, providing further evidence that distinct identities between MGE and CGE are less clear and appear to be merged in cKO mice. Eigenvector centrality is a measure of the influence of a node in a network. (**B**) UMAP plots of MGE clusters showing expression of early markers of PV or SST fate in *Gad2Cre-Arx* cKO and WT samples. Abbreviations: CGE, caudal ganglionic eminence; LGE, lateral ganglionic eminence; MGE, medial ganglionic eminence

Supplementary Figure 5

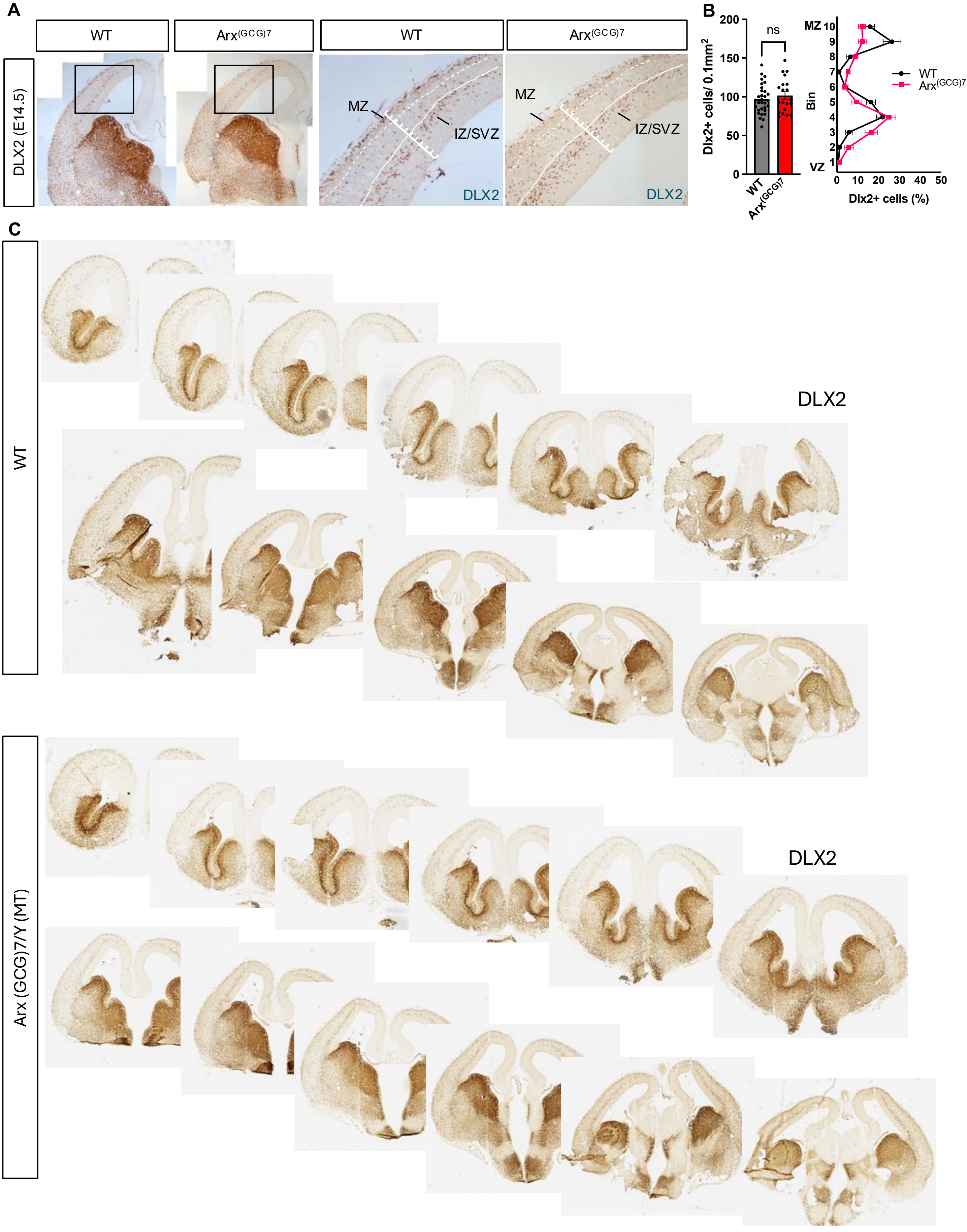

Supplementary Fig 5. cIN density and distribution defects in Arx(GCG)7/Y. (**A**) Representative images of DLX2 immunolabeling on coronal sections of the embryonic (E14.5) WT and Arx(GCG)7 forebrains. (**B**) Left: Quantification of the percentage of DLX2+ cells in each bin over the total number of DLX2+ cells in all 10 bins. Right: Quantification of tdTomato+ cell density. (**C**) Representative series of images of DLX2 immunohistochemical labeling (DAB based) on coronal sections of the Arx (GCG)7 or WT brains.

Supplementary Figure 6

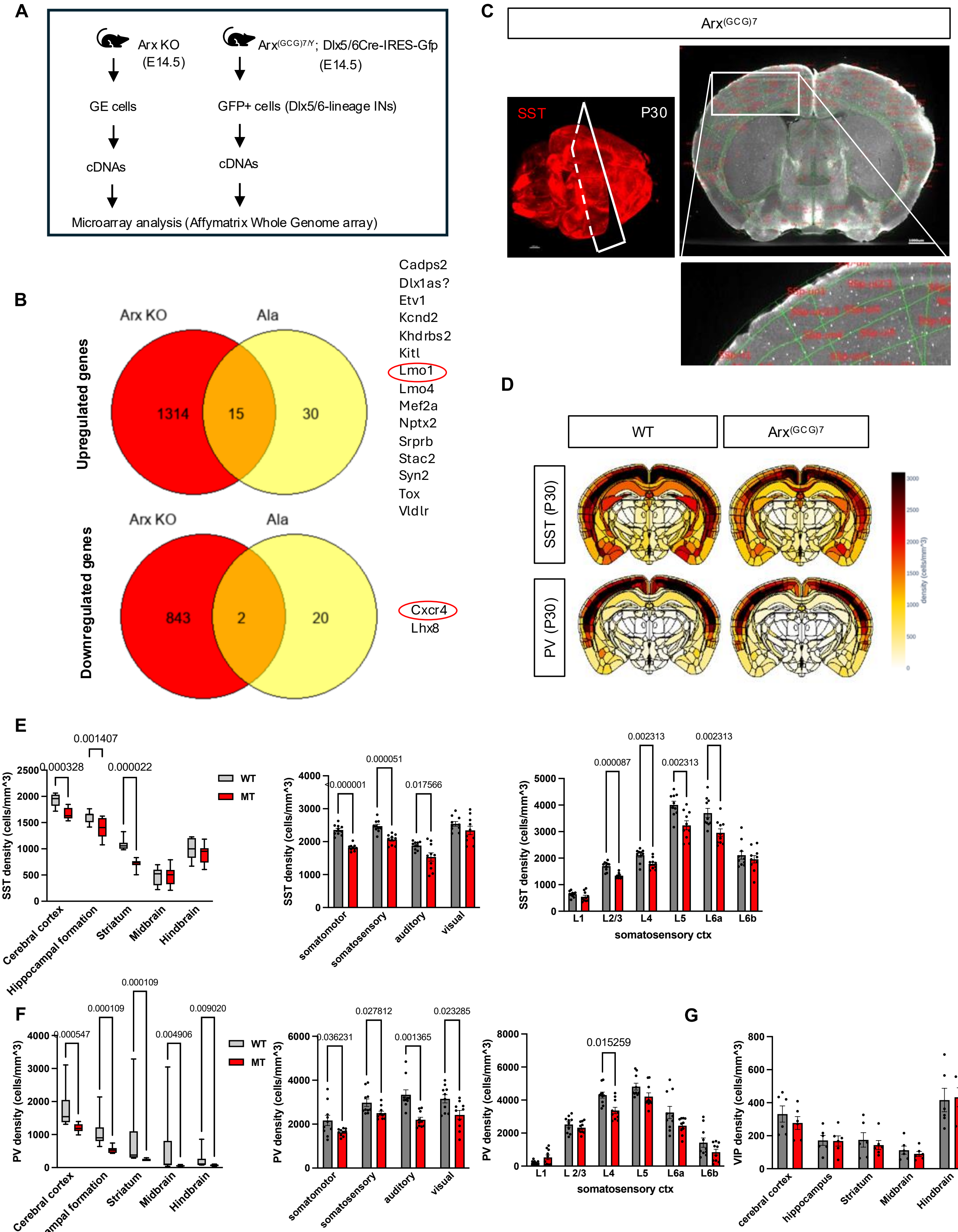

Supplementary Fig 6. (**A**) A summary schematic depicting microarray analysis in Arx(GCG)7; Dlx5/6CreGFP and Arx KO mice. (**B**) Differentially expressed genes common both in Arx(GCG)7 and Arx KO mice. Venn diagram of up and downregulated genes identified from RNA microarray analysis. (**C**) Representative analysis of 3D immunolabeling of SST on adult brains (P30) of Arx(GCG)7. Left: a dorsal view of the whole brain with a slice at the level of anterior hippocampus. Right top: An example coronal section (SST immunolabel) overlayed with reference brain atlas for quantitative analysis. Right bottom: Magnified image of the boxed area in top (red labels and green lines indicate each area annotated in the Alan Brain Atlas). (**D**) Heat maps on the coronal section illustrations showing the densities of the SST or PV immunolabeled cells of the WT and Arx(GCG)7 brains. (**E-G**) Quantification of SST (E), PV (F), or VIP (G) IN cell density (cells/mm^3) in each indicated brain regions, each cortical area, or each indicated layer of the somatosensory cortex of the WT and Arx$^{(GCG)7}$(labeled as MT) brains, processed for 3D immunolabeling with VIP antibody after tissue clearing procedure. Number over the bar: P value. Multiple unpaired t-test. Scale bars: 200 μm

Supplementary Figure 7

UMAP of Gad2 lineage cINs (ligands and receptors)

A

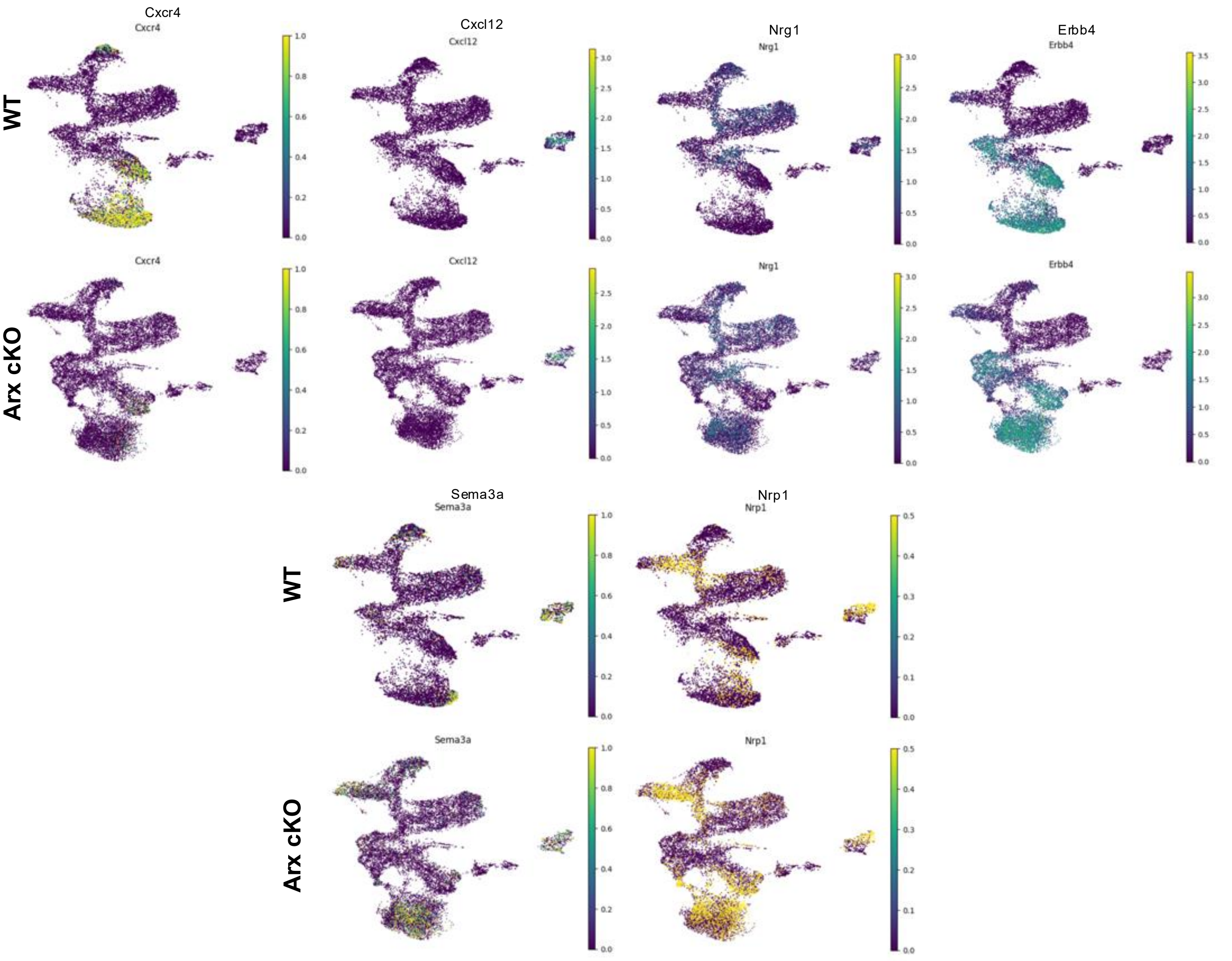

B

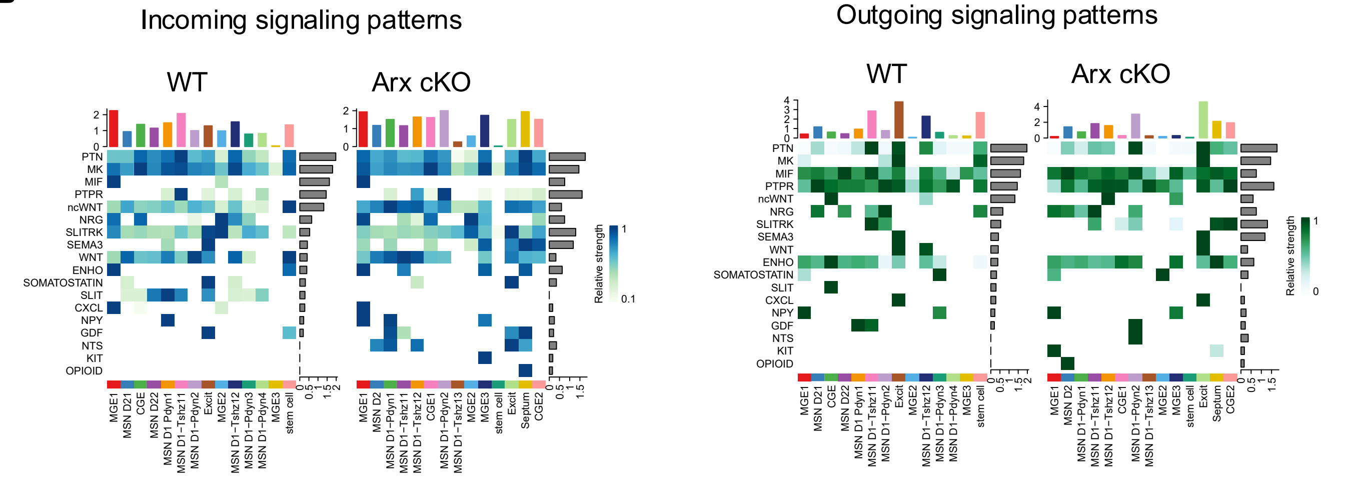

Supplementary Fig 7. (**A**) UMAP plot showing expression of ligands and receptors involved in IN migration cell migration in *Gad2Cre-Arx* cKO and WT cell clusters identified in scRNA-seq analysis. (**B**) Heatmaps displaying representative signals contributing to outgoing or incoming signaling of each cell group identified in scRNA-seq of WT and cKO.

Supplementary Figure 8

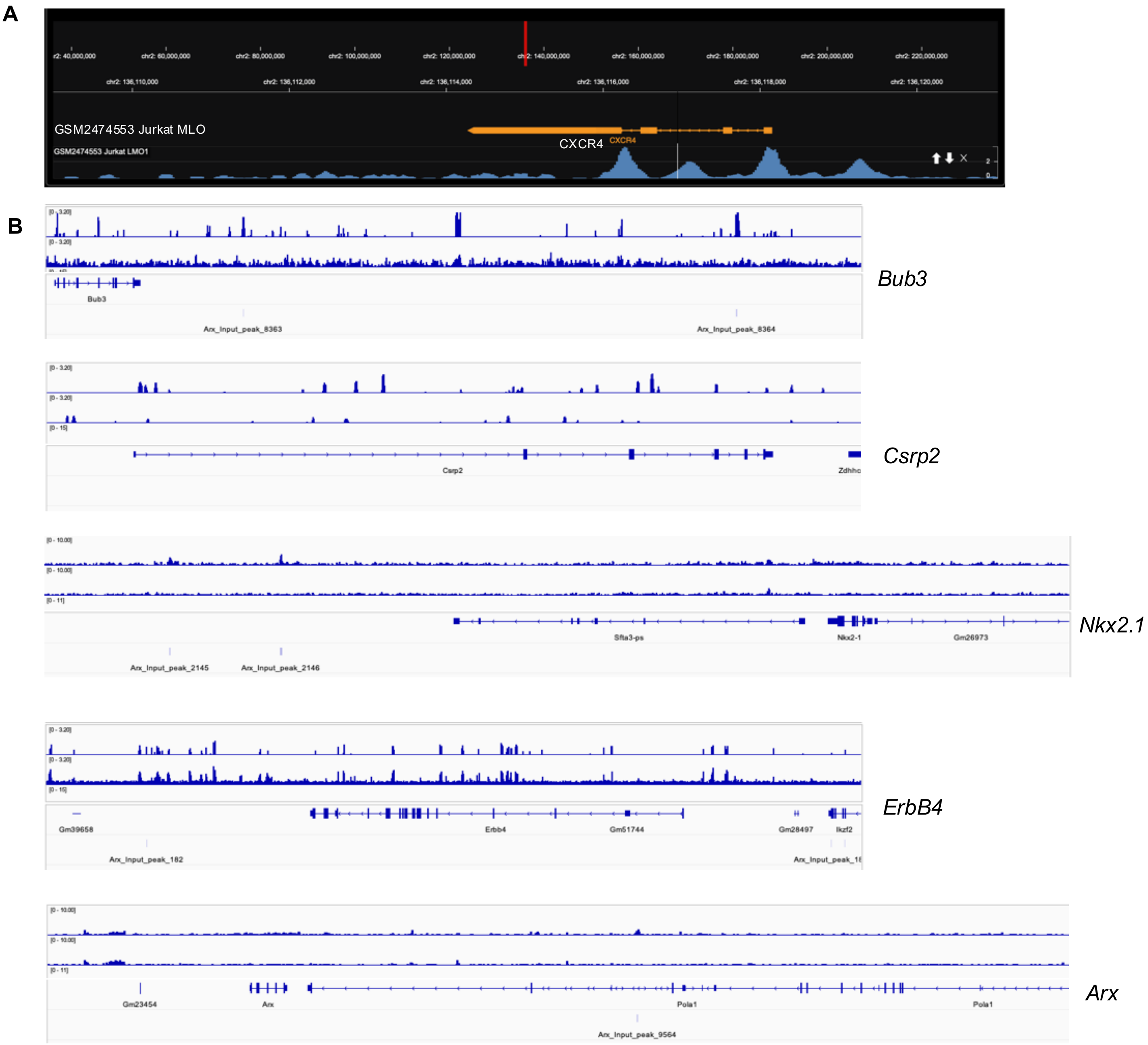

Supplementary Fig 8. **A** LMO1 ChIP-seq peak analysis showing specific LMO1 peak at *Cxcr4* locus (ref. [82]). **B** Examples of gene loci identified as ARX binding sites in ARX ChIP-seq analysis.

Supplementary Figure 9

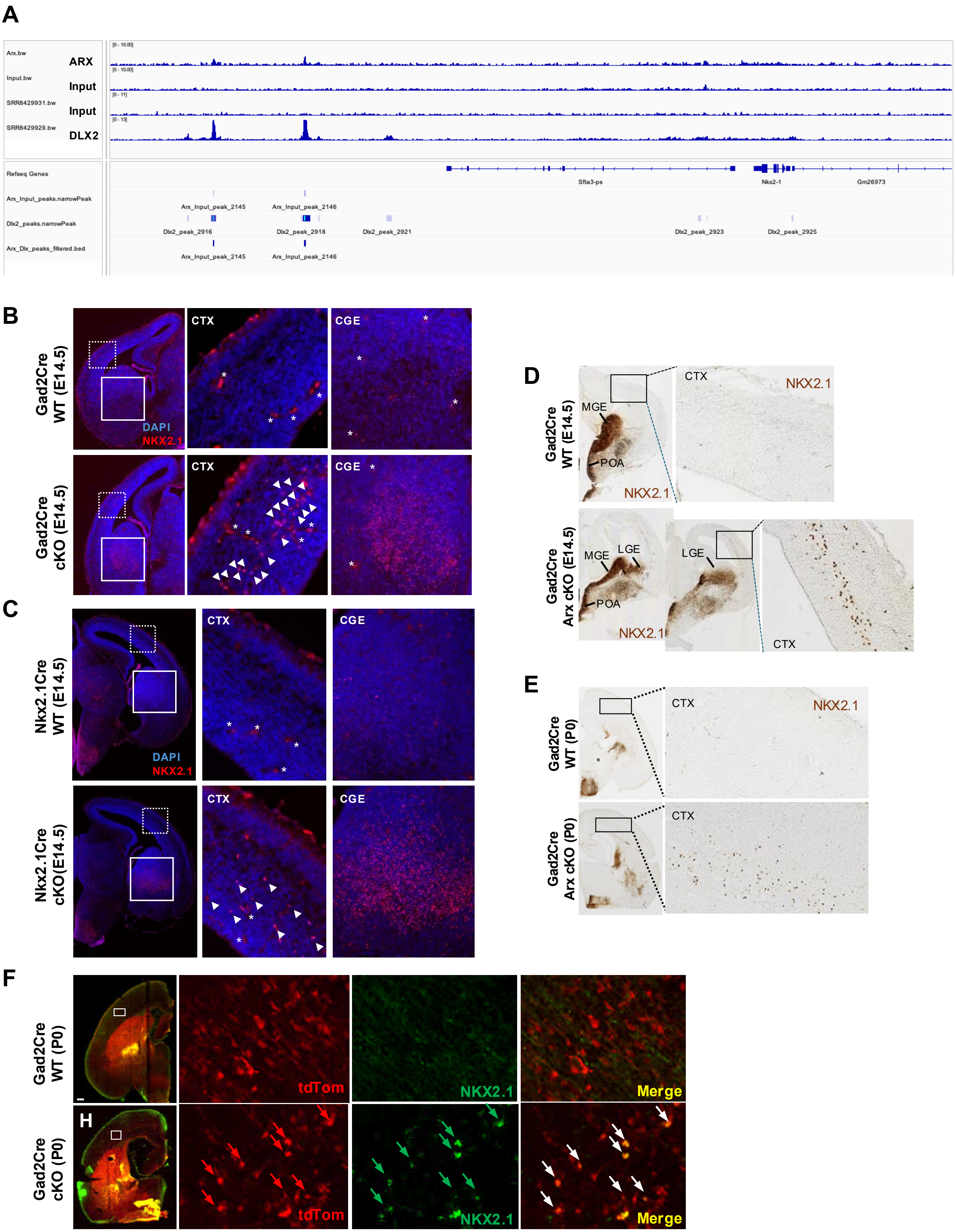

Supplementary Fig 9. (**A, B**) Representative images of NKX2.1 immunofluorescence on E14.5 coronal sections of the Gad2Cre-Arx WT vs. -Arx cKO (A) or Nkx2.1Cre-Arx WT vs. - Arx cKO brains (B). NKX2.1+ cells are shown as red; DAPI (blue), for nuclear staining; CTX and CGE panels are magnified images of the dotted (CTX) or solid boxed areas of the left panels; arrow heads, NKX2.1+ cells; asterisks, blood cells with autofluorescence. (**C, D**) Representative images of the coronal sections of the *Gad2Cre-Arx* cKO and WT brains at E14.5 (C) or P0 (D) with DAB-based immunohistochemical labeling of NKX2.1. (**E**) Representative images of NKX2.1 immunofluorescence with tdTomato on P0 coronal sections of the WT or Gad2Cre-Arx cKO brains. The right 3 panels are magnified images of the boxed area in the left panels. tdTomato+ (*Arx*-deficient, red) cells are shown in red; NKX2.1+ cells, green; DAPI (blue), for nuclear staining; red, green, and white arrows indicate tdTomato+, NKX2.1+, and double+ cells, respectively. Abbreviations: CGE, caudal ganglionic eminence; LGE, lateral ganglionic eminence; MGE, medial ganglionic eminence
